# Low effectiveness of influenza vaccines vis-à-vis mechanism of protection by vaccines – potential causes and recommendations to improve control of influenza

**DOI:** 10.3389/fimmu.2026.1816148

**Published:** 2026-05-20

**Authors:** Rajesh K. Gupta, Kishore R. Alugupalli, James L. Cowell

**Affiliations:** 1 Biologics Quality & Regulatory Consultant, LLC, North Potomac, MD, United States; 2 TurboVax Inc., Horsham, PA, United States; 3 Cowell Vaccine Consulting, Holly Springs, NC, United States

**Keywords:** annual vaccination, antigenic seniority, influenza antiviral drugs, influenza vaccines, low effectiveness, manufacture in eggs, monoclonal antibodies, non-egg-based influenza vaccines

## Abstract

Current licensed influenza vaccines primarily protect by eliciting antibodies against the viral hemagglutinin (HA) glycoprotein, thereby blocking viral attachment and fusion with host cells. Unlike most vaccines, influenza vaccines must be administered annually because circulating viruses undergo continuous antigenic drift and population antibody titers wane over time. Despite yearly reformulation, influenza vaccine effectiveness remains highly variable, often below 45%, largely due to antigenic mismatches. These mismatches arise from ongoing HA evolution following strain selection and from egg-adaptation during production or propagation in animal cell cultures, which can alter key HA epitopes relative to circulating strains. Even when an antigenic match is favorable, repeated annual vaccination may elicit immunological phenomena that attenuate protective responses. Serial vaccination in young and older adults can increase regulatory T-cell activation, reducing vaccine-induced antibody titers. In older adults, this may be compounded by age-associated CD4+ T-cell memory populations that have reduced capacity to activate HA-specific B cells. While natural influenza infection induces durable memory B cells, conventional vaccination does not reliably generate such long-lived memory, suggesting a fundamental limitation of current vaccine platforms. Collectively, these observations underscore the need to re-evaluate influenza vaccination strategies, particularly to improve protection in high-risk groups such as older adults. Reducing antigenic mismatch remains essential and can be facilitated by improving the vaccine selection process and expanding the use of recombinant protein and mRNA vaccine production platforms that do not rely on egg- or animal-cell culture technologies. In parallel, the rational selection and development of adjuvants that minimize T-regulatory cell induction while enhancing durable memory B-cell formation and long-lived plasma cells may help overcome the immunological constraints associated with repeated annual vaccination. Beyond active immunization, complementary countermeasures are critical for mitigating severe outcomes, including hospitalizations and deaths. Antiviral drugs and monoclonal antibodies, especially those engineered for extended *in vivo* half-life, represent important adjuncts for protecting vulnerable populations such as the elderly, young children, and immunocompromised individuals. Strengthening and advancing these modalities should be prioritized as part of an integrated strategy to improve influenza control and reduce the global burden of disease.

## 1 Introduction

Except for safe water, vaccines have been the most effective public health intervention in reducing mortality and morbidity due to many infectious diseases (1). One of the deadliest diseases in the history of mankind, smallpox, has been eradicated by universal vaccination with the smallpox vaccine (2). Poliomyelitis has been eliminated from the Western Hemisphere through effective vaccination campaigns (3). While measles was nearly eliminated, imported cases still pose a threat to unvaccinated individuals and those who are immunocompromised (4). Vaccines have also controlled many other diseases with high morbidity and mortality, including tetanus, diphtheria, pertussis, typhoid, mumps, rubella, chickenpox, shingles, yellow fever, diseases caused by hepatitis B, hepatitis A, human papillomavirus, Pneumococci, Meningococci, and *Haemophilus influenzae* type b. Vaccines benefit people of all ages, protecting the elderly and vulnerable individuals from infectious diseases and preventing infection-related cancers (5). They also reduce the risk of antimicrobial resistance (AMR) and the risk of transmitting disease to others in the community, thereby providing herd immunity.

**Figure 1:**
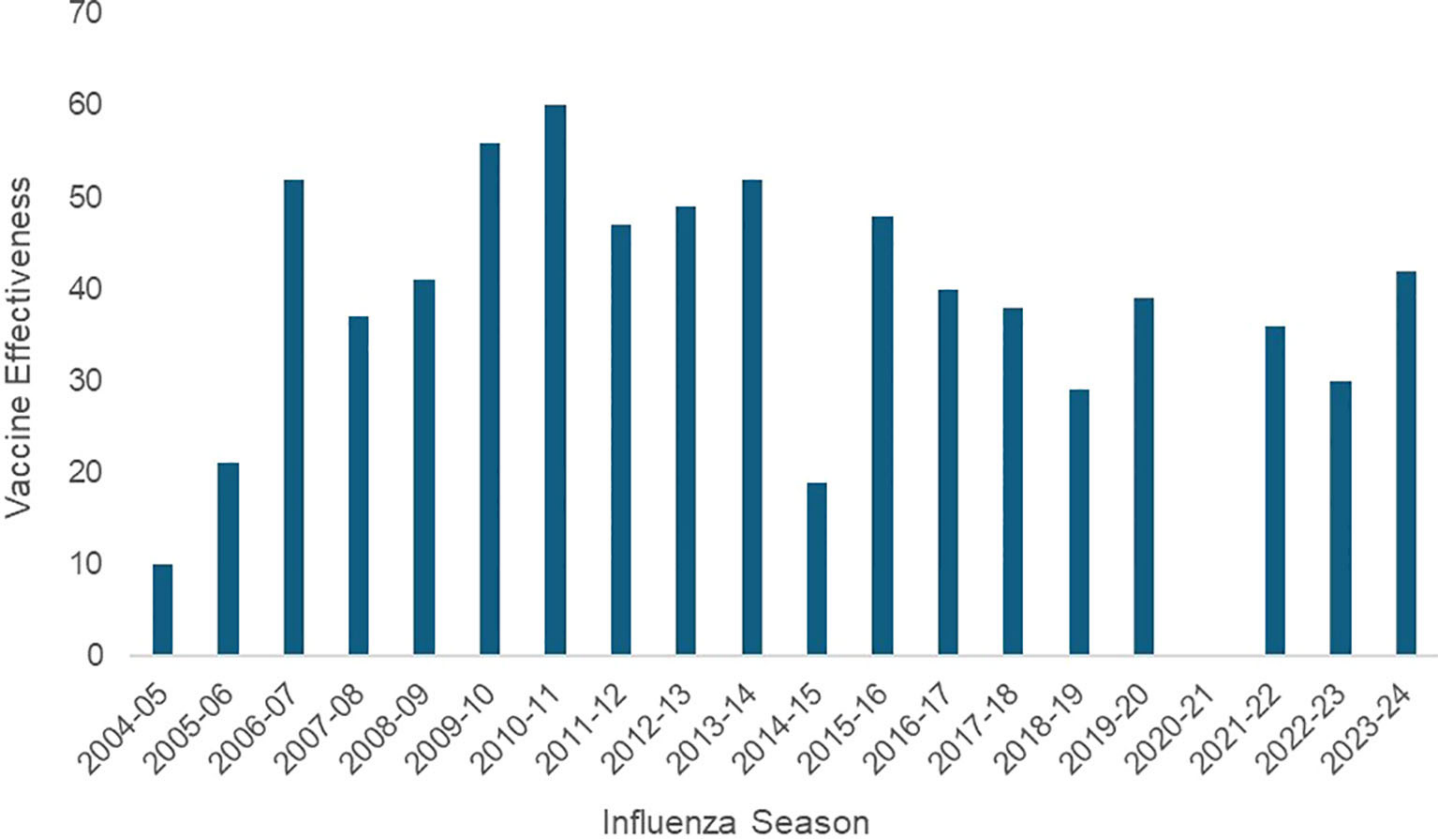
Adjusted effectiveness estimates of the annual influenza vaccine in the United States. Data from the Centers for Disease Control and Prevention (CDC). https://www.cdc.gov/flu-vaccines-work/php/effectiveness-studies/?CDC_AAref_Val=https://www.cdc.gov/flu/vaccines-work/effectiveness-studies.htm. Influenza vaccine effectiveness was not estimated for 2020–21 because of low influenza virus circulations during that season due to behavioral measures, such as social distancing and mask wearing for the COVID pandemic.

Despite the broad success of vaccines and the Centers for Disease Control and Prevention (CDC)’s recommendation for annual vaccination for all adults and children (6), influenza vaccines show low and variable effectiveness (7–11) (**Figure 1**). Developed in the mid-1930s, the first commercial influenza vaccines were approved in the United States in 1945 (12). Until the 2011–2012 influenza season, trivalent vaccines (covering two A subtypes, H1N1 and H3N2, and one B strain from either Victoria or Yamagata lineages) were used in the US. Due to frequent lineage mismatches between the B strain in the vaccine and circulating strains, which reduced the effectiveness of the trivalent vaccine against B viruses (13, 14), the World Health Organization (WHO) recommended adding strains from both B-lineage viruses for the 2012–13 influenza season. Following the apparent disappearance of the B/Yamagata lineage since 2020 (15, 16) due to lockdowns, social distancing, and other behavioral measures during the COVID-19 pandemic, the WHO recommended removing this component, leading to a global transition back to trivalent vaccines beginning in the 2024–2025 season (17).

Current trivalent inactivated influenza vaccines contain strains of two influenza A subtypes (H1N1 and H3N2) and an influenza B virus from the Victoria lineage and are given annually by parenteral intramuscular injection except for Flumist, which is given by intranasal spray. Influenza vaccines are administered annually due to waning antibody levels and the need to match the vaccine HA antigen to circulating wild-type strains, which evolve through seasonal antigenic drift driven by genetic changes. Since influenza viruses exhibit continual antigenic drift, the WHO recommends specific strains for annual seasonal vaccines in February for the Northern Hemisphere, subsequently approved by the US Food and Drug Administration (FDA) for the US influenza vaccine, and in September for the Southern Hemisphere (18–20). The WHO’s recommendations for strains depend on global viral surveillance data from the previous 5 to 8 months and occur 6 to 9 months before vaccine deployment (10, 18–20). Current vaccines are not very effective (18, 19, 21, 22), and a new virus strain often emerges that is not part of the seasonal vaccine (mismatch), significantly reducing the vaccine’s effectiveness during the annual influenza season. Since the 2004–05 season, adjusted influenza vaccine effectiveness has fluctuated between 10% and 60%, with 12 of these 19 years below 45% (**Figure 1**), and an even lower 95% confidence interval of protection (21). This uncertain vaccine effectiveness contributes to low public acceptance; in the 2023–24 season, vaccination coverage in the US was 55.4% among children (6 months–17 years old) and 44.9% among adults (≥18 years old) (22). The questionable effectiveness of influenza vaccines contributes to vaccine hesitancy and affects the public’s uptake of other vaccines.

This article aims to explore the immunological mechanisms underlying vaccine-induced protection, examine the factors contributing to the relatively low effectiveness of influenza vaccines (**Table 1**), and offer strategic recommendations to improve the control of influenza disease.

**Table 1:** Factors for low effectiveness of influenza vaccines.

Factor	Comments
Antigenic Drift	Small number of mutations in the Hemagglutinin (HA) and Neuraminidase (NA) antigenic sites
Antigenic Shift	Major changes in the antigenic types of HA and NA expressed due to genetic exchange in the segmented genome of influenza viruses
Design of Seasonal Influenza Vaccine	Estimation from circulating strains from the previous influenza season
Manufacturing Process	Effect of Egg adaptation, isolation, and passage or propagation in animal cell cultures on the receptor-binding site of vaccine strains
Original Antigenic Sin/Antigenic Seniority	Annual repeated vaccination
Repeated Vaccination	Low protection, lower antibody response, due to the generation of regulatory T-cells
Antigen used in Serological Methods	Use of egg-grown virus in hemagglutination inhibition (HAI)
Vaccination Complacency measures	False sense of protection avoiding preventive behavioral
Immune Response to Infection and Vaccination	Influenza infection elicits long-term memory B-cells, while no such response with vaccines

## 2 Immune response to vaccines

Understanding the mechanisms by which vaccines confer protection is essential for enhancing their effectiveness. The mechanism of protection by vaccines is complex. However, licensed vaccines, except for Bacille Calmette-Guerin and adenovirus vaccines, primarily confer protective immunity by eliciting serum IgG antibodies (23) against virulence factors of pathogens, including toxins secreted by pathogens, receptors that bind to host cells to initiate infection, and capsular polysaccharides that help evade phagocytosis. Known mechanisms by which antibodies confer protection include facilitating complement-mediated lysis or opsonophagocytosis of invading microorganisms, blocking pathogen-host cell binding or cell entry, neutralizing toxin activity (23), and mediating antibody-dependent cellular cytotoxicity (ADCC) against large parasites and pathogen-infected cells. Other potential protective mechanisms include cytotoxic CD8+ T lymphocytes, which recognize and destroy infected cells, thereby limiting the spread of infectious agents, and CD4+ T-helper (Th) lymphocytes, which contribute to protection by secreting specific antiviral cytokines and supporting the generation and maintenance of B-cell and CD8+ T-cell responses (24). Mucosal immunity, including IgA antibodies, also plays a role in protecting against infections and in preventing the transmission of infectious diseases. However, the mechanisms of mucosal immunity in protection are not fully understood, and there are challenges in developing strategies and methods to stimulate and measure it effectively (25–27).

### 2.1 Humoral immune response

Since all currently licensed influenza vaccines, except Flumist, are administered by intramuscular injection to provide protection primarily by stimulating hemagglutinin (HA) specific antibodies, it is important to review how the vaccine antigens are processed immunologically after injection. Such an understanding is important to devise vaccine formulations that could improve their effectiveness.

**Figure 2:**
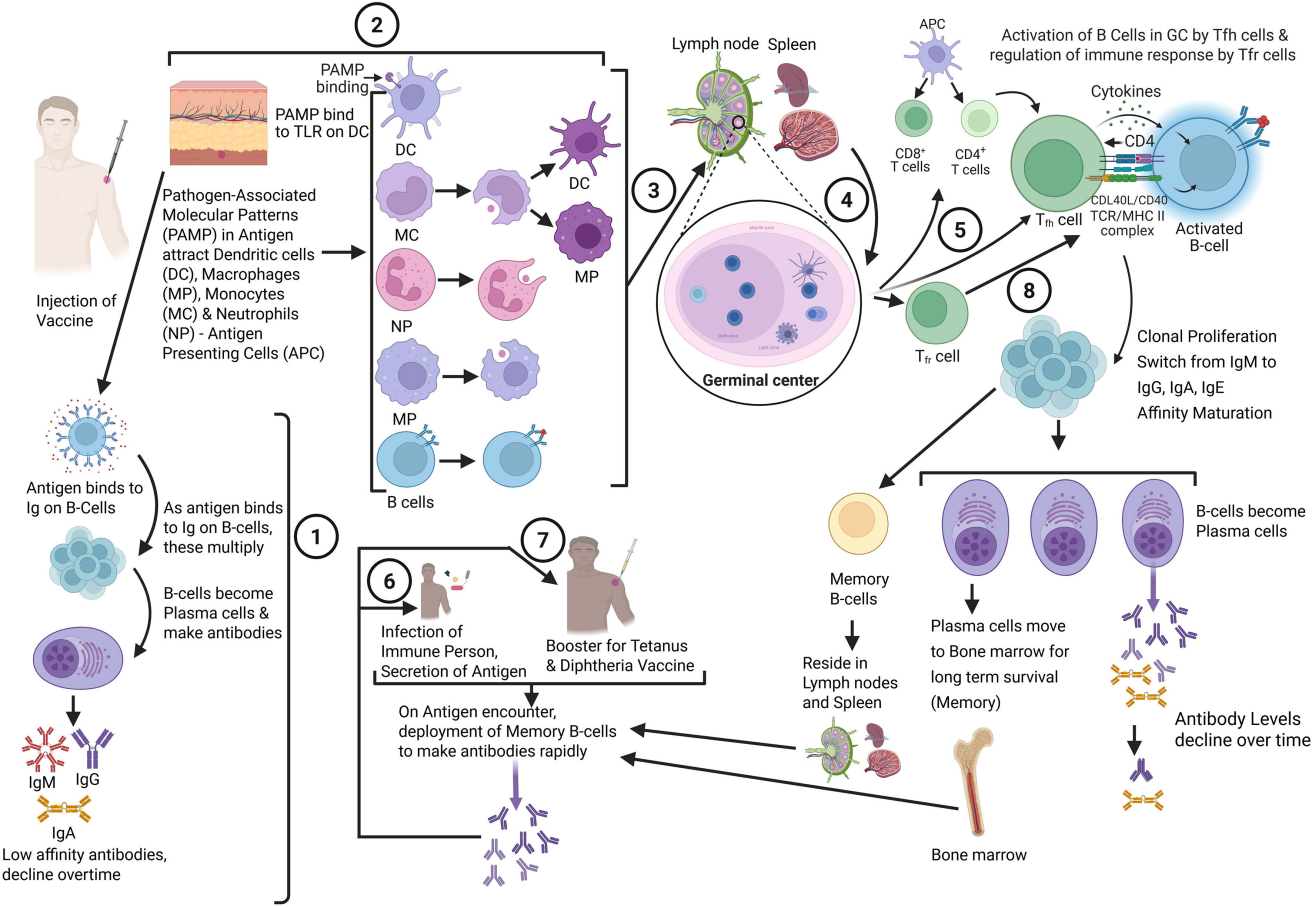
Humoral response in humans after the injection of vaccines. **①** Extrafollicular Response. Upon antigen injection, it migrates to lymph nodes or the spleen, where it binds to the B cell antigen receptor. Upon antigen recognition, the B cells undergo brisk activation and differentiate into plasma cells that produce low-affinity and short-lived antibodies, IgM ± IgG/IgA, at low levels in the serum within a few days after immunization. ② Germinal Center (GC) Response. Simultaneously, following the injection of an antigen, pathogen-associated molecular patterns (PAMPs) in the antigen attract antigen-presenting cells (APCs), including dendritic cells (DCs), macrophages (MPs), monocytes (MCs), and neutrophils (NPs), that patrol the body. MCs can further differentiate into MPs or DCs. These APCs engulf the antigen, get activated through “danger signals” from PAMPs, and migrate to the draining lymph nodes and/or spleen ③, where the activation of T and B lymphocytes takes place. Antigen-specific T-helper (Th) cells are activated by antigen-bearing DCs, triggering antigen-specific B cells and initiating the GC reaction ④. In GCs, B cells receive additional signals from follicular T cells (Tfh) ⑤ and undergo clonal proliferation; switch from IgM to IgG, IgA, or IgE antibodies and affinity maturation; and differentiate into plasma cells secreting large amounts of high-affinity antigen-specific antibodies. A few plasma cells exit lymph nodes and/or spleen and migrate to the bone marrow, for long-term memory (years to decades). On leaving GC, some B cells, instead of differentiating into antibody-secreting plasma cells, differentiate into memory B cells that transiently migrate through the blood toward the extrafollicular areas of spleen and lymph nodes. These memory cells persist there as resting cells until re-exposed to their specific antigens. ⑥ On re-exposure to the specific antigen, either by antigen secreted by the pathogen infecting the person, or by booster doses, memory B cells rapidly proliferate and differentiate into plasma cells secreting large amounts of high-affinity antibodies within a few days after infection or the booster dose, clearing the infection in the person, ⑦ or maintaining high levels of circulating antibodies, as for tetanus and diphtheria vaccines. ⑧ Within the GC, a specialized subset of Tregs, known as T follicular regulatory (Tfr) cells modulate the magnitude and quality of the antibody response. In essence, Tfh and Tfr cells form a balanced system in the GC, where Tfh cells provide “help” and Tfr cells provide “control, “ ensuring a high-affinity yet regulated antibody response. Created in BioRender. Gupta, R. (2026) https://BioRender.com/085abl7.

When a protein or other antigen is injected, it reaches the lymph nodes or spleen, where B cells bind to it through B cell antigen receptors (i.e., surface immunoglobulins), which get activated and differentiate into plasma cells, producing antibodies within 7–14 days of immunization. This initial extrafollicular response results in the rapid appearance of low-affinity IgM and low IgG antibodies (**Figure 2**) (24, 28), which are short-lived because these plasma cells have a short lifespan; antibody levels decline rapidly and eventually return to baseline. Simultaneously, the pathogen-associated patterns in the injected vaccine antigens attract circulating dendritic cells (DCs), macrophages, monocytes, and neutrophils, activating these through “danger signals” (24). This activation alters their surface receptor expression, including major histocompatibility complex (MHC) molecules, and induces their migration to the draining lymph nodes, where T and B lymphocytes are activated. Antigen-specific CD4+ helper T cells, activated by antigen-presenting DCs, induce antigen-specific B cells to migrate towards follicular dendritic cells (FDCs), initiating the germinal center (GC) reaction, a transient microenvironment that lasts for a couple of weeks post-antigen exposure (**Figure 2**) (29, 30). Within GCs, B cells receive further signals from follicular helper T cells (Tfh), leading to extensive clonal expansion of antigen-specific B cells, antibody class switching (from IgM to IgG, IgA, or IgE), affinity maturation, and differentiation into memory B cells and plasma cells that secrete high amounts of antigen-specific antibodies, typically peaking around 4 weeks post-immunization (24). The magnitude and quality of vaccine-induced humoral immunity are determined within the GC through iterative interactions between B cells and Tfh cells (31).

Another subset of T cells that plays a role in regulating the immune response is regulatory T cells (Tregs) (32–34), which are essential for maintaining self-tolerance and preventing autoimmunity, with the majority originating in the thymus as tTregs. Within the GC, a specialized subset of Tregs, known as T follicular regulatory (Tfr) cells, which differentiate from these Tregs precursors, specifically modulate the magnitude and quality of the antibody response. Tfh and Tfr cells act as opposing forces within GCs to control antibody responses. In essence, Tfh and Tfr cells form a balanced system in the GC, where Tfh cells provide “help” and Tfr cells provide “control, “ ensuring a high-affinity yet regulated antibody response (35).

### 2.2 Importance of memory B cell response

Memory B cells, generated in response to T cell-dependent antigens during the GC reaction alongside plasma cells, do not immediately differentiate into antibody-secreting cells upon leaving GCs (24, 29, 36). These memory B cells transiently migrate through the blood to the extrafollicular areas of the spleen and lymph nodes (**Figure 2**), where they remain as resting cells until they re-encounter their specific antigens. Typically, a single immunization results in a sub-optimal immune response. However, during a secondary immune response, for example, following a booster dose or an encounter with the antigens produced by the pathogen on infection, reactivation of antigen-specific memory B cells results in rapid proliferation and differentiation into long-lived plasma cells, secreting large amounts of high-affinity antibodies that are detectable in serum within days of antigen exposure (24). While short-lived plasma cells generated by extrafollicular response maintain peak antibody levels for a few weeks before declining rapidly, the long-lived plasma cells generated by follicular response (GC response) reside in bone marrow survival niches and continue to produce antigen-specific antibodies, albeit with a slower decline (24).

Circulating antibodies and memory B-cells elicited by prior infection or vaccination are crucial for protection against infectious diseases. IgG has a half-life of 21 days; to maintain sufficient and durable levels needed for protection, it needs to be produced continuously. Over time, antibody levels decline, sometimes falling below the estimated protective levels, likely due to a reduction in memory B cells and plasma cells. The protective levels of circulating antibodies are unknown for most diseases. Despite low circulating antibody levels for extended periods, most vaccines provide long-term or lifelong protection without the need for booster doses, except for toxin-mediated diseases such as tetanus and diphtheria. Protection against most diseases is not necessarily achieved by maintaining high circulating antibody levels. Instead, it is through the rapid deployment of the immune system after infection, which activates memory B cells in response to antigens expressed by invading pathogens (**Figure 2**). However, maintaining protective antibody levels is crucial for preventing toxin-mediated diseases such as tetanus and diphtheria. This is achieved through periodic booster doses of tetanus-diphtheria (Td) or tetanus-diphtheria-pertussis (TdaP) vaccines to adults every 10 years.

### 2.3 Importance of cellular immune responses

Following uptake of antigens by professional antigen-presenting cells, such as DCs, the antigens are processed into small peptides and displayed on their cell surface in MHC molecules for presentation to T cells (24, 37). CD4+ T cells, with an antigen-specific T cell receptor (TCR) and matching MHC II-peptide specificity, are activated, providing further activation signals to DCs and differentiating into effector cells that primarily produce Th1 or Th2 cytokines, which shape B cell and other cell-mediated immune responses. For example, annual influenza vaccination can generate populations of Tregs (38), specifically Tfr cells, in all age groups (39) and CD4+ memory T cells in the elderly, which can diminish protective antibody responses by downregulating B cell activation (40). CD8+ T cells recognize MHC class I-peptide complexes and differentiate into cytotoxic effector cells capable of directly killing infected cells by releasing cytotoxic granules into the infected cell.

## 3 Immune responses to influenza vaccines

Influenza infection and vaccines primarily elicit antibodies against the major surface glycoproteins, hemagglutinin (HA) and neuraminidase (NA) (41). Antibodies targeting HA and NA play crucial roles in mediating protection against reinfection. Since most influenza vaccines primarily contain the HA antigen with traces of NA, the immune response after vaccination is predominantly directed against HA (41). HA antibodies from infected and recovered individuals, as well as hyperimmune equine antiserum, have been used to treat influenza patients. During the 1918 influenza pandemic, convalescent human serum transfer reduced the absolute risk of death in patients from 26% to 8% when administered within 4 days of symptom onset (12), demonstrating the role of HA antibodies in protection against influenza. Crowe (42) suggested using antibodies, particularly a combination of monoclonal antibodies, as biological drugs to prevent or treat influenza infection. The potential use of specific monoclonal antibodies to control influenza is discussed in section 5.3 below.

Regulatory agencies recognize Hemagglutination Inhibition (HAI) titers as the primary correlate of protection for influenza vaccine efficacy and use this data to approve influenza vaccines (42). HAI titers >1:40 are traditionally associated with a 50% protection rate (43). However, other studies have shown that these target titers are not accurate predictors of protection, particularly in children and the elderly, and often overestimate the level of protection (44–46). HAI titers >1:40 as a correlate of protection against influenza are fundamentally flawed for live-attenuated influenza vaccine, FluMist (47), and non-egg-based influenza vaccines. It is important that appropriate HAI titers and/or other correlates of protection, such as virus neutralization titers or anti-NA immunity, be considered based on the population (children or elderly) and vaccine platform to evaluate the effectiveness of influenza vaccines. Further comments on the use of the HAI serological assays and the role of anti-NA immunity in protection against influenza are presented in section 4.6 below.

Typically, circulating serum antibodies to viral and vaccine antigens persist for varying durations, ranging from years to decades. Antibody responses following viral infections or live vaccines have half-lives of 50 years or more, while responses to nonreplicating protein antigens (tetanus and diphtheria) are lower, with estimated half-lives ranging from 11 to 19 years (48). The persistence of influenza-specific antibodies is short-lived after vaccination (42, 49–52), which justifies the need for annual vaccination, in addition to the evolution of new influenza strains driven by antigenic drift, which is discussed in more detail in sections 4.1 and 4.2 below.

Vaccination with the same strain is recommended in consecutive influenza seasons when circulating viruses show no meaningful antigenic change. There have been several instances of the same influenza strain in more than one influenza season, notably A(H1N1) pdm09. Following its emergence in 2009, the H1N1 vaccine component remained unchanged for several years (A/California/7/2009 and immediate successors) until 2017, when antigenic drift in the 6B lineage prompted a change to A/Michigan/45/2015 (53). Further antigenic evolution led to subsequent updates (notably A/Brisbane/02/2018, A/Victoria, and A/Wisconsin lineage picks) to address reduced vaccine effectiveness against emerging subclades, as documented by laboratory serology and real-world vaccine effectiveness studies (54, 55). Despite the continued use of H1N1 and related strains in annual influenza vaccines for over 15 years, the A(H1N1)pdm09 strain and its new subclades have remained widely circulating. It remains unclear whether repeated annual vaccination with antigenically similar A(H1N1)pdm09 strains provides substantial protection against the disease, as A(H1N1)pdm09 infections continued in subsequent years. During the 2015–2016 influenza season, an increased prevalence of A(H1N1)pdm09 influenza virus infection was observed in Israel (56). More recently, A(H1N1)pdm09 was the predominant influenza A virus circulating in the United States during both the 2023–2024 and 2024–2025 seasons (57, 58), again raising questions about the degree of protection conferred by the vaccine component. This may be due to changes in the HA-binding site of strains grown in eggs and/or to repeated annual vaccination triggering Tfr cell-induced immunological tolerance mechanisms, as referenced and discussed in this article in section 4.5.

Furthermore, these observations suggest that repeated annual vaccination with the H1N1 strain for over 15 years does not induce a long-lived circulating memory B-cell response. In contrast, memory B cells following natural influenza infection are known to be remarkably long-lived. In 2007, Crowe and investigators isolated B cells from nearly 100-year-old subjects that neutralized the 1918 H1N1 and related early 20^th^-century H1N1 influenza viruses, including those that had not circulated in the human population for approximately 5 decades (59–62). This persistence of B cell memory exhibited unexpected protection during the 2009 H1N1 pandemic, which contained antigenic elements of the 1918 virus, in the elderly compared to those without prior exposure to early 20^th^-century H1 viruses (63–65). The same type of immunity has been observed with H3 viruses in humans exposed to older influenza strains that entered the human population in 1968 (42). From these observations, it appears that memory B cells often remain in circulation for decades (or even a lifetime) following influenza infection, protecting against related strains encountered many years later in life, but probably not against those induced by vaccination.

Cellular immune responses have been shown to play a crucial role in protection against influenza, and current inactivated split-virus vaccines do not elicit or stimulate robust cellular immunity (66). Cytotoxic T-cell immunity was found to be more broadly cross-reactive against related influenza strains than humoral immunity (67). It is possible that future influenza vaccines that stimulate viral neutralizing antibodies to HA and NA, in addition to stimulating cytotoxic T-cells to conserved viral proteins expressed on the surface of infected cells, will provide much improved effectiveness compared to currently used vaccines.

As discussed in sections 4.1 and 4.2 below, protective epitopes on HA and NA in circulating strains are highly variable due to antigenic drift and antigenic shift. Studies using human monoclonal antibodies, followed by structural and functional characterization, revealed conserved antigenic sites recognized by broadly cross-reactive antibodies (42). HA has two major structural domains with protective antigen sites: the head and the stem. The design of “headless” stem antigens has served as the basis for several current “universal influenza” candidate vaccine programs (42), but with limited success. Nonetheless, stem antibodies showed broad cross-reactivity, including recognition of groups 1 and 2 influenza A viruses, as seen with the stem antibody FI6 (68). Many stem antibodies demonstrated lower potency in virus-neutralization tests than head-domain antibodies, and the protective and therapeutic effects of many stem antibodies in preclinical animal models have been associated with antibody-dependent cellular cytotoxicity (ADCC) activity (69). Even if HA stem-cell-based vaccines may stimulate ADCC activity, the inability to elicit strong viral neutralizing antibodies suggests that they would be insufficient as standalone prophylactic or therapeutic vaccines.

Antibodies targeting the globular head domain of HA, particularly those directed against the receptor-binding site (RBS), exhibit the most neutralizing or HAI activity against influenza viruses. RBS is the immunodominant region of the HA head domain because its structural elements are highly exposed on the virion surface and readily accessible to B-cell receptors, thereby facilitating antibody recognition (42). RBS is a structural domain comprising a shallow pocket surrounded by hypervariable loops and helices. Many of the amino acids at this site are highly conserved to maintain the sialic acid receptor-binding site that binds to human cells to initiate infection. Several key residues that interact with sialic acid are conserved across influenza A and B HAs (70). However, four features surrounding the RBS, including the 130-loop, 150-loop, 190-helix, and 220-loop, which are designated by their relative positions in the HA amino acid sequence, are hypervariable. This mutability is responsible for the antigenic drift observed over time in influenza.

A highly effective immune response, both humoral and cellular, may not be elicited by current influenza vaccines due to repeated vaccinations (see section 4.5), the phenomenon of original antigenic sin (OAS) or antigenic seniority (see section 4.5.1), and mismatches between the HA of the vaccine and circulating strains. In addition to antigenic drift, the selection procedure of influenza strains for manufacture and isolation (see sections 4.2 and 4.4), as well as the growth of influenza viruses in eggs (sections 4.3) and perhaps in animal cell culture (section 4.4), contribute to mismatches between the HA of vaccine and circulating strains. In addition to eliciting antibodies to conserved epitopes through OAS or antigenic seniority, repeated vaccination can induce immune tolerance and increase the number of HA-specific Tfr cells, which are a specialized subset of Tregs defined by co-expression of FOXP3 and CXCR5, enabling their migration into B cell follicles where they co-localize with GC B cells (33, 71, 72). Within this niche, Tfr cells impose multilayered control over GC dynamics through both contact-dependent and cytokine-mediated mechanisms, including CTLA-4–dependent modulation of co-stimulatory signals and suppression of Tfh cell help, as well as the production of immunoregulatory cytokines such as IL-10 and TGF-β (73–76). Through these mechanisms, Tfr cells constrain GC magnitude, regulate somatic hypermutation and affinity maturation, and limit plasma cell differentiation and antibody output (74–79). Emerging evidence from immunization models further indicates that the magnitude and timing of the Tfr response critically shape vaccine-induced humoral immunity, such that early or excessive engagement, such as repeated immunization of this regulatory axis, can dampen GC responses and reduce the magnitude and durability of antibody production (79–81). Collectively, these findings provide a mechanistic framework supporting the concept of vaccine “blunting, “ or “attenuated magnitude”, wherein heightened Tfr activity attenuates the breadth, magnitude, and persistence of antigen-specific antibody responses.

As discussed in section 4.5, repeated annual vaccination generates Tregs, specifically Tfr cells, in all age groups (39, 82) and CD4+ memory T cells in the elderly, which can diminish protective antibody responses (40). These cells can suppress the immune system’s ability to fight off influenza infections, affecting both humoral and cellular responses, including the failure to elicit memory B cells, which are primarily responsible for the long-term protection provided by vaccines. Moritzky et al. (83) observed a negative association between preexisting HA-specific antibodies and postvaccination antibody levels. Furthermore, preexisting CD4+ T cell levels were negatively correlated with vaccine-induced CD4+ T cell expansion. Thus, although memory CD4+ T cells and serum antibodies consist of components that can enhance vaccine responses, the accumulated immunity specific to influenza A H1 and H3 proteins is associated with diminished future responses (83).

## 4 Factors for low effectiveness of influenza vaccines

### 4.1 Antigenic drift and shift

The major challenge in preventing influenza is the development of strain-specific vaccines. The high rates of mutations (antigenic drift) and genetic reassortment (antigenic shift) in influenza viruses are vital to the virus’s ability to cause seasonal epidemics and occasional pandemics, respectively. Influenza viruses continually evolve to evade recognition by the host immune system, acquiring mutations in the antigenic sites of HA and NA. This process is referred to as “antigenic drift”, which is the primary reason for annual influenza vaccination, in contrast to other vaccines, except the COVID-19 vaccine, where annual boosters are not typically required after completing the primary vaccination series.

The influenza virus genome is segmented, which allows genetic material to be exchanged when two strains of influenza A virus infect the same host cell in human or animal reservoirs, including birds, pigs, horses, and bats (84–87). This exchange alters the transcribed HA and/or NA antigens, potentially producing a new influenza A virus subtype to which humans are not immune (86). This process, known as antigenic shift, has led to influenza pandemics. Continuous antigenic variation, whether through antigenic drift or antigenic shift, is a significant limitation in developing a broadly protective, universal influenza vaccine.

### 4.2 Antigenic drift and vaccine design timelines contribute to annual seasonal influenza vaccine mismatch

Antigenic drift refers to the virus’s ability to evade pre-existing immunity. Antigenic drift is the slow, gradual accumulation of small genetic mutations in a virus, such as influenza, that change its surface proteins (antigens), making it harder for the immune system to recognize it and leading to seasonal epidemics and the need for annual influenza vaccines. Antibodies to an influenza variant in the population, whether acquired through vaccination or infection, eliminate that variant, allowing new antigenic variants to become predominant (12). When antibodies to the latest variant are elicited, yet another antigenic variant emerges in the population, and the cycle continues. This ongoing process of antigenic drift ensures a continually renewed pool of new variants infecting susceptible hosts, even among those who are vaccinated.

**Figure 3:**
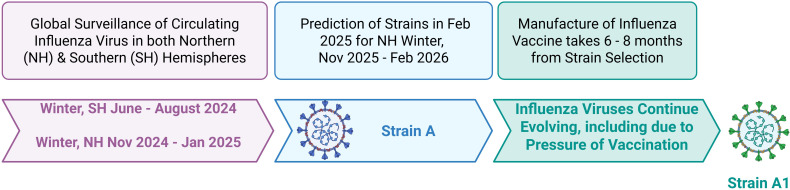
Design of annual influenza vaccine, with a hypothetical example for the 2025–26 influenza season for one of the three strains. For example, Strain A is selected for the 2025–26 Influenza Season; during vaccine manufacturing, Strain A changes to Strain A1, resulting in a mismatch between the vaccine and circulating influenza virus strains. Created in BioRender. Gupta, R. (2026) https://BioRender.com/2rgjunf.

Strains for annual influenza vaccines for the northern and southern hemispheres are projected based on global surveillance of circulating influenza strains from the prior influenza season in the opposite hemisphere (**Figure 3**) (18–20). Strain selection and the egg-based vaccine manufacturing process take 6–9 months. However, antigenic drift continues in nature during this timeframe and even after annual vaccinations begin, leading to a “mismatch” between the vaccine and circulating strains (**Figure 3**). This significantly reduces vaccine effectiveness (10). For example, during the 2014–2015 influenza season in the United States, more than 80% of the circulating influenza A (H3N2) viruses differed from the vaccine virus, and vaccine effectiveness was only 13% against influenza A (H3N2) (10); the 2017/18 trivalent vaccine which had a low effectiveness of ∼25% in the UK was due to mismatch to the predominant influenza A strain and to the circulating Yamagata strain (85, 88), the latter was not in the 2017/18 trivalent vaccine. The dominance of the A/H3N2 subclade K during the 2025–2026 season (89–92) and its absence from this season’s influenza vaccines further demonstrated deficiencies in vaccine strain selection and manufacturing processes for influenza vaccines, where the vaccine strain was no longer optimally matched to the viruses circulating during the 2025–2026 season, leading to low influenza vaccine effectiveness.

### 4.3 Role of manufacturing process – egg adaptations of influenza viruses cause antigenic differences between vaccine and circulating influenza strains

**Figure 4:**
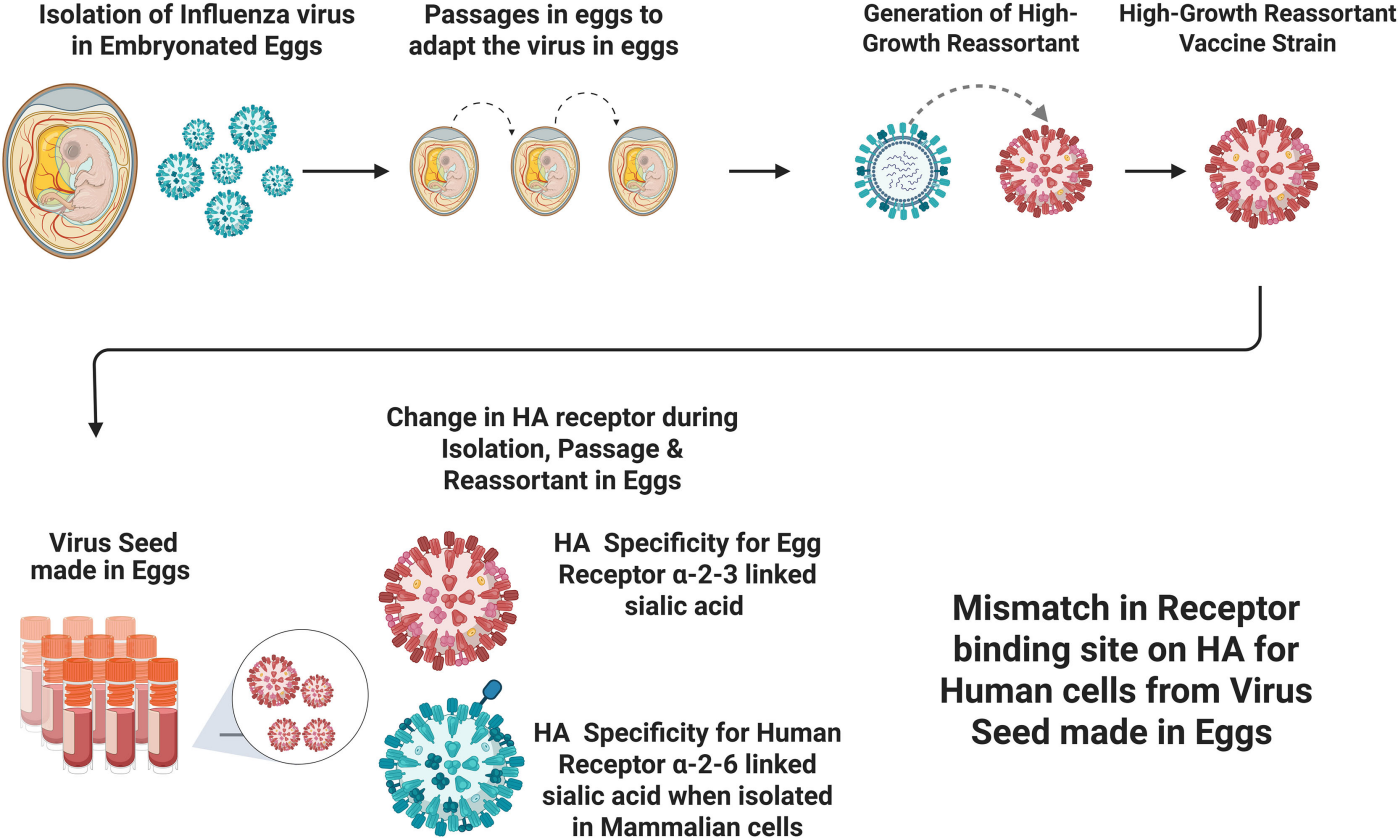
The manufacturing process of influenza vaccine in embryonated eggs with an adverse effect on the Hemagglutination (HA) binding site on the vaccine strain as compared to the same circulating strain. Created in BioRender. Gupta, R. (2026) https://BioRender.com/cv2cqtx.

Most influenza vaccines (approximately 80% or more) are manufactured in embryonated eggs, with one vaccine, Flucelvax, produced in cell culture using Madin-Darby canine kidney (MDCK) cells and another, Flublok, containing recombinant HA produced in baculovirus-infected insect cells (12, 93). Influenza virus strains for the manufacture of vaccines, both in eggs and cell cultures, are isolated in embryonated chicken eggs (**Figure 4**) or primary chick kidney cultures to provide a barrier to extraneous agents that might originate in the clinical source material (12). One of the reasons for isolating influenza viruses in embryonated eggs was to prevent contamination of influenza vaccines with adventitious or extraneous agents from clinical source material (94), because extraneous agents do not propagate in eggs but do in mammalian cells. This was particularly important due to the abbreviated timelines for manufacturing annual influenza vaccines and the lengthy testing required to detect extraneous agents. This should not be a concern with advances in rapid detection of extraneous agents in cell substrates used in the manufacture of biological products (95), and if influenza vaccines are not given annually, as recommended in this article.

Virus isolates are further passaged to adapt them for growth in eggs, a process necessary for producing influenza vaccines made using egg-based technology (**Figure 4**). Thereafter, reassortants of “high growth” influenza A viruses are generated for their maximal replication in eggs, facilitating vaccine manufacture. These reassortants combine the HA and NA from virus strains selected for the annual seasonal influenza vaccine with the high growth properties of the donor virus, A/Puerto Rico/8/34 (PR8), a strain adapted to replication in eggs (96, 97).

This process of isolation, passage, and generation of reassortants of virus strains in eggs results in critical amino acid substitutions, leading to differences in key epitopes on HA between vaccine and epidemic or circulating strains (98). There are differences in RBS on HA between human and avian cells: the influenza virus HA RBS is α-2, 6-linked sialic acid for human cells and α-2, 3-linked sialic acid for avian cells found in eggs (**Figure 4**) (98–101). RBS-directed antibodies elicited by influenza vaccines recognized the egg-adapted H1 strain, but not the circulating 2009 pandemic virus (101). Egg-adaptation mutations (e.g., T160K, L194P) create highly accessible decoy epitopes that establish an immunodominance hierarchy. These decoys steer the B-cell response toward non-protective sites and away from the functionally conserved RBS pocket. This redirection of antibody specificity ultimately erodes vaccine effectiveness against circulating wild-type strains (102–105).

Katz and Webster (106) reported that primary isolation of type A influenza (H3N2) virus in mammalian cell cultures, including MDCK cells, monkey kidney LLC-MK2 cells, human lung continuous cell line MRC-5, and primary guinea-pig kidney cell culture, resulted in a virus with HA identical to that of the virus replicating in the infected individual. In contrast, similar isolation of the virus in the embryonated eggs resulted in the selection of variants with amino acid substitutions in the globular head region of the HA molecule (106).

Schild et al. (107) demonstrated that cultivating influenza B viruses in eggs produced subpopulations antigenically distinct from those grown in mammalian cell cultures. Consequently, the selective environment during the manufacturing of egg-derived influenza vaccines leads to mutations in the HA protein, which may elicit antibodies that do not bind to circulating influenza strains, thereby reducing the effectiveness of the vaccines (101, 107–118). For example, Zost et al. (119) demonstrated that the site B of H3N2 HA, T160 HA glycosylation mutation was missing in the 2016–2017 egg-propagated vaccine strain and present in the circulating wild virus strain (49, 119). This change resulted in both ferrets and humans exposed to the egg-adapted A/H3N2 vaccine strain producing poorly neutralizing antibodies to the circulating A/H3N2 viruses during that season. A recombinant HA vaccine with the T160 HA glycosylation was superior to the egg-adapted and passaged H3N2 virus, and the observed probability of influenza-like illness was 30% lower in subjects who received the recombinant vaccine than in subjects who received the inactivated influenza vaccine (49, 120). Thus, the lower efficacy of egg-adapted vaccine viruses may have resulted from mutations or changes in the A/Texas/50/2014 vaccine virus, leading to low vaccine effectiveness.

Another example of the mismatch between the circulating strain and the egg-adapted vaccine strain is from the 2012/2013 influenza season, which resulted in only 46% efficacy in adults aged18 through 49 years and 9% efficacy in people older than 65 years of age (121). In this case, the circulating strain prediction was correct, but the mismatch was due to a mutation in the egg-adapted seed virus (IVR-165) that was sent to vaccine manufacturers. The mutation was not present in the WHO-recommended strain but occurred during adaptation of the strain to grow in eggs. The lower efficacy (9%) in older adults may also be due to age-related reprogramming, leading to a functional Th2 cell bias in memory T cells that has been linked to dysregulated B cell responses against highly boosted antigens in influenza vaccines (40) and the generation of Tfrs after repeated vaccination (39, 82).

An extensive literature search and expert panel study (122) concluded that there is a mechanistic basis for reduced vaccine effectiveness resulting from variation in candidate influenza viruses due to egg-based manufacturing. Overall, the study’s evidence suggested that, despite being an established method for producing influenza vaccines worldwide, egg-based manufacturing likely reduced vaccine effectiveness because the virus changed during adaptation to eggs. This effect was believed to be particularly pronounced against certain strains, such as the influenza A/H3N2 virus, possibly due to a higher mutation rate in this strain (123).

As discussed, it is well documented that egg-based manufacturing can alter the gene and amino acid sequences of HA, resulting in a mismatch between the HA protein in the vaccine and that in circulating viral strains, and such mismatches are correlated with a reduced effectiveness of annual influenza vaccination. However, continuous drift in influenza viruses after selecting virus strains for manufacture would still result in reduced effectiveness of non-egg-based vaccines (discussed in section 4.2 and **Figure 3**).

### 4.4 Role of manufacturing process – animal cell culture adaptations of influenza viruses cause antigenic differences between vaccine and circulating influenza strains

Since clinical influenza vaccine strains can be amplified in cell culture for strain selection and an influenza vaccine, Flucelvax, is manufactured using animal cell culture, MDCK, it would be important to determine whether growth in animal cell culture can also result in genetic and antigenic changes in HA. As discussed above, Katz and Webster (106) found that primary isolation of type A influenza (H3N2) virus in mammalian cell cultures yielded a virus with HA identical to that of the virus replicating in the infected individual. However, in a recent study, Prasai et al. (124) demonstrated genetic and antigenic changes in HA when A/H3N2 clinical strains were grown in a humanized MDCK cell line. They evaluated the genetic sequence and antigenicity of HA from 45 A/H3N2 clinical isolates using techniques (Next-Generation Sequencing, NGS, and a Poly (lactic acid), PLA antigenicity assay) that did not require prior expansion in eggs or cell culture before analysis. These assays were then applied to the A/H3N2 strains after growth in MDCK cells. The results showed that isolation of clinical strains using MDCK cells yielded considerably different HA sequences and antigenic forms than those found in direct analyses of circulating human strains. The authors concluded that, to improve the selection of influenza vaccine strains for inclusion in annual vaccination formulations, direct analysis of clinical isolates would be preferable to analysis after cell culture expansion (124). This would also apply to the use of eggs for isolation and passage in vaccine strain selection.

Prasai et al. (124) did not evaluate the effects of serial passage of A/H3N2 in MDCK cells, which is required for the manufacture of commercial-scale annual vaccines. However, it is likely that such a passage would further support the selection of strains with HA antigenic forms different from those of clinically circulating A/H3N2 strains. Also, the authors did not conduct experiments with influenza strains other than A/H3N2. Still, these data are very relevant to the design of annual influenza vaccines, as Flucelvax is produced using MDCK cell culture and A/H3N2 strains have been major circulating strains during recent influenza seasons, including the 2025–26 season (89–92).

These limitations due to passage in cell culture and egg-based vaccines can be overcome with new vaccine manufacturing technologies, especially recombinantly expressed purified proteins (93) and mRNA vaccines (125–128). A recent study of health care workers aged 21–49 years after receiving quadrivalent influenza vaccines (93), the recombinant Flublok vaccine, the cell-culture-based Flucelvax, and the egg-based Fluarix during the 2022–2023 season found that the 14-day post-vaccination HAI titers against cell-grown A/H3N2 were 2.9-fold higher for Flublok than for Flucelvax and Fluarix. The HAI titers were not different between Flucelvax and Fluarix. Nine of the total ten cases and all five A(H3N2) cases were among vaccinees who received Flucelvax and Fluarix (93). It is not clear whether the increased HAI titers and better protection afforded by Flublok, compared with those obtained for Flucelvax and Fluarix, were due to HA antigenic changes in the wild-type circulating A/H3N2 strain during growth in cell culture and eggs or a higher vaccine dose, 45 µg of HA for Flublok versus 15 µg for Flucelvax and Fluarix. It is possible that the immunological benefit of Flublok was due to its higher dose or to its HA structure that might differ from that of Flucelvax and Fluarix, which were modified during passage in MDCK cells and eggs.

Further, shorter manufacturing time for recombinantly expressed purified proteins and mRNA vaccines, 3–4 months and 2–3 months, respectively, compared with 6–8 months for current egg- and cell culture-based vaccines, may help better predict the strains that will be prevalent in the upcoming influenza season. There are recent proposals supported by data (19) that using HA genetic sequences from wild-type strains obtained closer (e.g., 3 months) to the start of the influenza disease season could provide a better match between the vaccine HA strain and the circulating strains for that season. This would be especially important for strains like A/H3N2, which appear to have a higher mutation rate (123). However, for current egg- and cell-culture-based vaccines, strains need to be selected at least 6 months before the start of the influenza season to ensure that commercial vaccine lots are available.

### 4.5 Effect of annual vaccination (repeated vaccination) on protection and antibody response

Unlike other vaccines, for which annual booster doses are typically not required after completing the primary vaccination series, influenza vaccination is recommended annually. The need for annual vaccination has been justified due to the evolution of influenza viruses through antigenic drift and a reported decrease in antibody levels (49–52). Circulating influenza viruses continuously evolve in the human population and mutate under pressure from antibodies to the HA (12), leading to antigenic drift. However, vaccine effectiveness often decreases with repeated yearly influenza vaccinations. There are numerous scientific reports on the reduction in influenza vaccine effectiveness after repeated vaccinations (49, 120–122, 129–159), starting with a 1970s vaccine trial in an English boarding school, which observed that infection rates were higher among boys vaccinated in the current and previous seasons than among those receiving their first vaccination (160). These observations from three outbreaks of influenza A in a school suggested that annual revaccination with inactivated influenza A vaccine confers no long-term advantage (160). When the vaccine strain for the annual influenza vaccine is not updated and the circulating virus strain antigenically drifts away from it, negative interference associated with repeated vaccination appears to be exacerbated (145).

Immune responses to the influenza vaccine also decline among older adults due to immunosenescence (49). However, modeling studies have suggested that poor vaccine effectiveness in older age groups is better explained by repeated vaccination than by age-associated immunosenescence (161). In a recent study, Gong et al. (40) found that three annual vaccinations with quadrivalent vaccines containing the same influenza B strain (B/Phuket) since 2015 reduced HAI titer in a much higher percentage of 55 to 65-year-olds than in younger adults aged 25 to 35 years. HAI titers to other influenza strains were reported to be similar across age groups. Reduction in HAI titers to the highly B/Phuket-boosted HA antigen was associated with the generation of altered CD4+ memory T cells in older adults with reduced ability to activate antibody-producing B cells. Another important factor that could contribute to the low effectiveness of influenza vaccines is that repeated annual vaccination can generate Tregs, specifically Tfrs, across all age groups (39, 82), which can diminish protective antibody responses. The presence and ability of Tfrs and CD4+ memory T-cells to diminish the activation of antibody-producing B-cells could pose a challenge to improving the effectiveness of annual influenza vaccines, especially in adults >65 years old. This is important because it can occur even when there are good antigenic matches between the vaccine and circulating wild-type viruses.

Serological studies also showed reduced post-vaccination antibody levels in individuals who had received multiple annual influenza vaccinations compared to those who received a single influenza vaccination (162–169). Fox et al. (169) suggested that the ability of yearly vaccination to update immunity against new influenza viruses might be limited by pre-existing immunity. Thompson et al. (166) found higher antibody titers against A(H3N2) in participants who received a single vaccination than in those who received repeated annual vaccinations. Significantly lower antibody responses with repeat versus single vaccination were observed up to 18 months, suggesting a prolonged, blunted immune response beyond a single season (170). Sullivan et al. (171) observed decreasing post-vaccination antibody titers with increasing numbers of prior vaccinations in a cohort of Australian healthcare workers vaccinated with southern hemisphere quadrivalent vaccines in 2020 and 2021. The magnitude of the response to immunization, measured as the absolute titer or the rise in titer, was consistently higher in the group with no prior vaccination than in the group with at least five prior vaccinations. Antibody responses might increase upon encountering an antigenically distinct vaccine antigen, but infection was more immunogenic than vaccination (172). In a recent study (93), in health care workers aged 21–49 years during the 2022–2023 season, HAI titers in frequent vaccinees (three or more annual vaccinations during the last 5 years) showed lower HAI titers than those in infrequent vaccinees (zero or one vaccination during the last 5 years) for Flucelvax and Fluarix vaccines, but not for Flublok. Eight of the ten cases were detected among frequent vaccinees, and all five A(H3N2) cases were among frequent vaccinees who received Flucelvax and Fluarix.

The evidence discussed above suggests that repeated annual vaccination with the same HA antigens can reduce vaccine effectiveness by diminishing B-cell activation and the production of high-titer functional antibodies. Furthermore, the ‘immunological pressure’ from frequent vaccination in young adults might accelerate antigenic drift by promoting the emergence of vaccine-induced antibody-resistant strains.

#### 4.5.1 Repeated vaccination and the concept of original antigenic sin or antigenic seniority

**Figure 5:**
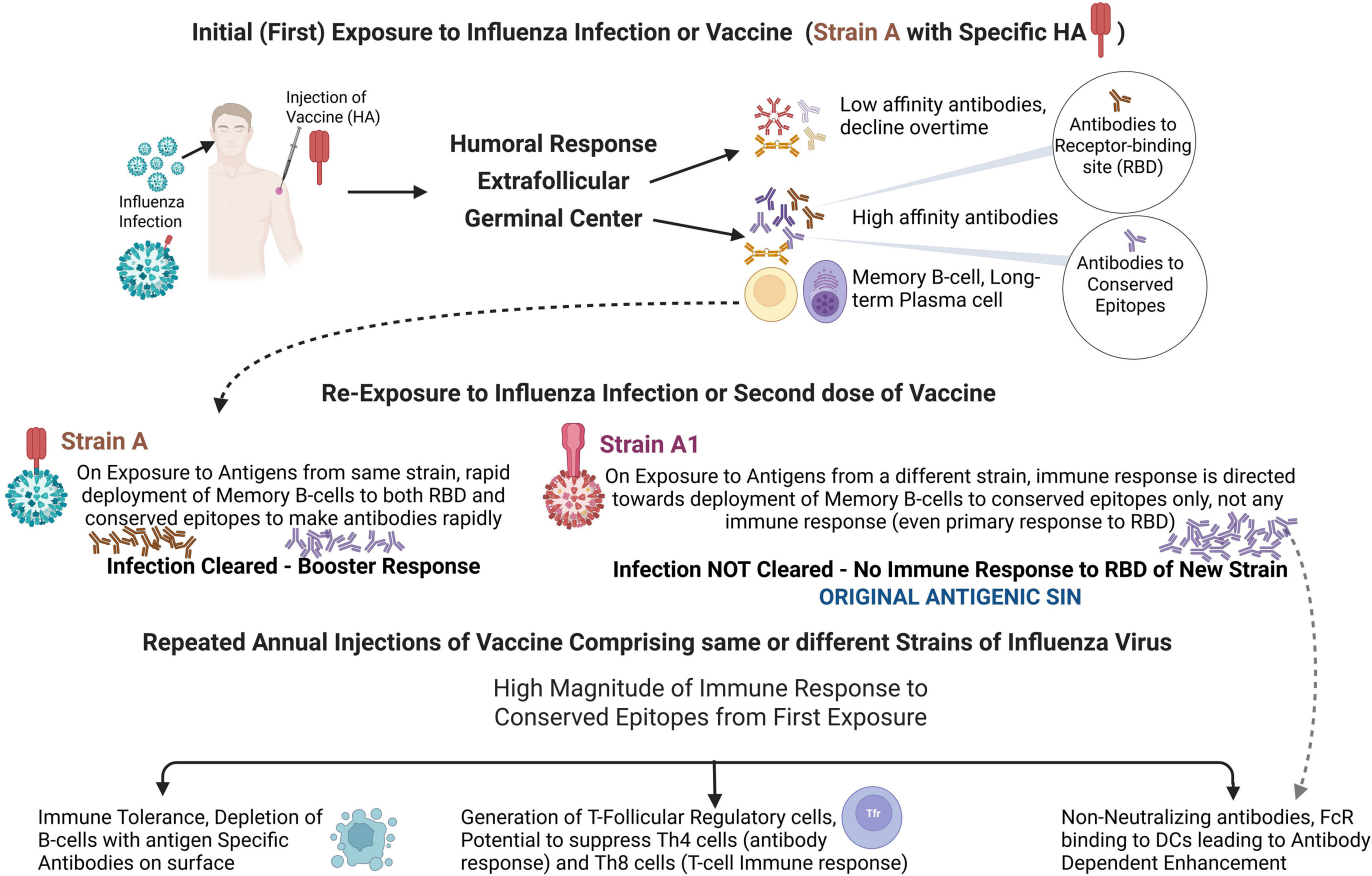
Projected immune response to influenza infection and vaccines. The effects of re-infection and repeated vaccination on the immune response, including the original antigenic sin phenomenon, the possibility of immune tolerance, the generation of T-follicular regulatory (Tfr) cells, and the induction of antibody-dependent cellular cytotoxicity, all of which affect protection from vaccination. Gupta, R. (2026) https://BioRender.com/5yhu0bp.

The concepts of OAS and antigenic seniority are central to understanding immune imprinting in influenza and its implications for vaccine performance after repeated vaccination. First articulated by Thomas Francis Jr. colleagues (173, 174) in 1960, OAS describes the propensity of the immune system to preferentially recall antibody responses elicited by the first influenza exposure when subsequently encountering antigenically drifted strains, thereby biasing responses toward conserved epitopes and potentially limiting the generation of *de novo* immunity to novel variants (**Figure 5**). While this classical view emphasizes immune interference, more recent work has refined the concept through the framework of antigenic seniority, which posits a hierarchical and cumulative pattern of antibody boosting in which responses to earlier (“senior”) exposures are preferentially amplified over those to later (“junior”) strains (175, 176). This model better reconciles longitudinal sero-epidemiological data, suggesting that immune history does not simply constrain responses but organizes them in a graded manner that can confer both cross-protection and variability in vaccine effectiveness across age cohorts.

Together, these paradigms underscore that prior antigenic exposures critically shape the magnitude, specificity, and breadth of responses to influenza vaccination, with important implications for strain selection, repeated vaccination strategies, and the design of next-generation or universal influenza vaccines. The concept of OAS suggests that prior infections have cumulative adverse effects on responses to later strains, resulting in higher antibody levels against strains encountered earlier in life (147). This is because antigenic drift does not alter the entire molecular structure of the HA or NA glycoprotein; cross-reactivity to non-drifted regions remains. Therefore, memory B cells to original common epitopes are preferentially upregulated over the generation of plasma cells and memory B cells to new epitopes, thus providing little to no protection against new variants (**Figure 5**). This process is believed to cause narrower immune responses after repeated vaccinations to tiny portions of the common antigen, leaving an individual with limited or no immune response to new epitopes on the emerging virus strain (52, 175, 177).

In a recent article, Zhang et al. (178) demonstrated that OAS occurs because CD4+ T cell proliferation and regulation signals are antigen-nonspecific. Rapidly responding memory CD4+ T cells trigger regulatory Tfr responses, which prematurely suppress naïve CD4+ T cell responses, leading to a similar OAS effect in CD4+ T cells (**Figure 5**). These findings indicate that the immune response is weakest at an intermediate level of cross-reactivity, a key characteristic of OAS, and may also help explain the role of OAS in antibody responses (178). Klenerman and Zinkernagel (179) demonstrated that OAS impairs cytotoxic T lymphocyte (CTL) responses to viruses bearing variant epitopes, thereby impairing the clearance of variant viruses.

However, the OAS phenomenon can enhance influenza immunity. During the 2009 pandemic, it was noted that older people had lower rates of infection than the younger population due to historical cross-immunity to conserved antigens from prior H1N1 viral infections (which were common in the late 1970s); thus, their original antigenic exposure offered protection (63–65). Based on these observations, it may be inferred that immune memory to influenza infections is long-term, and that repeated vaccinations are not beneficial in protecting against emerging strains.

Another concept, the “antigenic distance hypothesis, “ suggests that repeated homologous annual influenza vaccination with the same vaccine, despite increasing antigenic distance between an epidemic strain and the original vaccine strain, can lead to lower antibody levels and increased vaccine failure rates (51). This phenomenon, along with the OAS, is a vital factor in vaccine failure rates (51, 65).

### 4.6 Serological methods to evaluate humoral immune response after influenza vaccination

HAI and neutralization tests are commonly used to measure levels of strain-specific antibodies (12). However, the HAI test is most used to measure antibody response to influenza vaccines. In the HAI test, antibodies in an immune serum compete with chicken or turkey red blood cells (RBCs) in binding to the viral HA, thereby inhibiting the virus from agglutinating the RBC. In the absence of antibodies, viruses agglutinate red blood cells from chickens or turkeys. The neutralization test measures functional antibodies capable of neutralizing strain-specific influenza viruses and may be more sensitive than the HAI test (12). The usefulness of these methods in estimating the immune response to circulating influenza viruses depends on how the virus used in the test was grown, whether in eggs or cell cultures, so that the virus used in the method has a similar HA to that of circulating viruses. Virus grown in eggs is often used in the HAI test (43, 180–187), thus evaluating immune response to the mutant HA with a different epitope than that on the circulating influenza virus. In a study directly comparing HAI titers using an egg-grown vaccine versus an MDCK-propagated HA antigen, titers for 50% protection ranged from 203 to 437 for the egg-grown vaccine HA antigen and from 22.4 to 44.7 for the MDCK-propagated HA antigen, indicating a significant overestimation of HAI titers with the egg-grown vaccine antigen (187). Antibody responses determined to egg-grown antigens were higher than those to the corresponding cell-grown antigens, even among vaccine-naïve individuals (171). A strong response to egg-grown antigens may not provide a reliable correlate of protection against circulating viruses. Sullivan et al. (171) cautioned in interpreting immunogenicity studies performed using egg-grown antigens for the HAI test. The egg-grown virus in the HAI test measures HAI antibodies to egg-based vaccines, which may not be protective, rather than to circulating influenza viruses, reflecting protection. A few studies growing the viruses in MDCK cells for the HAI test used the original virus isolated and propagated in eggs.

Anti-NA immunity also contributes significantly to protection against influenza (188–191). However, to evaluate the effectiveness of current influenza vaccines, neither anti-NA immunity nor the NA content of these vaccines is measured during approval and release. Eichelberger and Monto (191) described that NA is largely ignored in the formulation and standardization of current influenza vaccines, primarily due to the lack of an easily performed test to measure NA antibodies. NA-specific immunity conferred broader protection against antigenic drift variants or newly emerging viruses that carry the same NA but a different HA subtype. Weiss et al (189) also found that NA inhibition titers associated with protection had a greater breadth of reactivity to drifted strains than virus neutralization titers (190). Targeting the immune response to both HA and NA may be critical for achieving optimal protection, since HA and NA elicit protective immunity through different mechanisms (188).

## 5 Recommendations for improving the control of influenza

The persistent low effectiveness of annual influenza vaccination necessitates a critical re-evaluation of current policy. This is important due to deficiencies in current vaccines and vaccination policies, supported by extensive scientific evidence, including the adverse effects of original antigenic sin, the mismatch between the vaccine and circulating strains resulting from the strain selection process, from egg- and animal cell culture based manufacturing, and the generation of HA-specific Treg or Tfr cells that can suppress the immune system’s ability to produce HA-specific protective antibodies. The vaccination strategy and policy for influenza vaccines require significant changes, along with alternative methods to control influenza infections, including the use of existing and development of improved antiviral drugs, passive immunization of high-risk patients with a cocktail of monoclonal antibodies, and the development of better vaccines capable of eliciting memory B cells. New vaccine antigenic targets should be selected from circulating human strains without prior amplification in animal cell culture or eggs, as discussed in section 4.4, and the vaccine antigens then produced using recombinant protein production systems and/or mRNA methods. In addition to these recommendations, behavioral measures that reduce exposure to respiratory pathogens also need to be implemented and better promoted.

### 5.1 Behavioral measures

Respiratory disease preventative behavioral measures that include mask-wearing, hand washing, and social distancing or contact avoidance are also important to utilize as they will not only protect against influenza but also against other respiratory viral diseases, including COVID-19, respiratory syncytial virus infection, the common cold, and emerging human metapneumovirus (hMPV) infections. This was evident during the COVID-19 pandemic, with lower influenza cases and a shorter annual influenza season (192–194), as shown in **Figure 1**, when few cases of influenza occurred during the 2020–21 influenza season due to behavioral measures, such as social distancing and mask-wearing, implemented during the COVID-19 pandemic. Interestingly, after lockdowns and behavioral measures implemented during the COVID-19 pandemic, the influenza B virus lineage Yamagata has not been detected since 2020, which is considered almost extinct, and has been removed from annual influenza vaccines (15, 16).

To manage the current threat posed by the influenza A(H3N2) Subclade K virus, nonpharmaceutical interventions, such as social distancing, have been suggested to reduce the burden of this season’s anticipated epidemic (89). Such measures should be voluntary, not mandatory. Individuals with symptoms of respiratory illness, including sore throat, runny nose, fever, body aches, etc., as well as their contacts, should practice behavioral measures to prevent the spread of respiratory infections during the winter months. The practice of such behavioral measures by healthy individuals in crowded places and by healthcare professionals is also recommended. Such behavior measures need to be better promoted by public health institutions and societies.

### 5.2 Influenza antiviral medications

Antiviral medications active against influenza viruses are essential in controlling influenza, particularly when influenza vaccines are ineffective. The CDC recommends four FDA-approved antiviral drugs for treating influenza infections (195), including Baloxavir, which was approved in 2022 for pediatric use in children aged 5 years and older.

- Oseltamivir phosphate (available as a generic version or under the trade name Tamiflu^®^)
- Zanamivir (trade name Relenza^®^)
- Peramivir (trade name Rapivab^®^)
- Baloxavir marboxil (trade name Xofluza^®^)

Antiviral medications are most effective when administered within 1 to 2 days of the onset of influenza symptoms. Influenza antiviral drugs can lessen symptoms and shorten the illness by about a day. Antiviral treatment shortly after symptoms can also help reduce some influenza complications. For adults hospitalized with influenza, some studies have reported that early antiviral treatment can reduce the duration of hospitalization and the risk of death (154). A recent study demonstrated that influenza antiviral treatment was underutilized among children and adolescents (196). All hospitalized children and adolescents, as well as those at higher risk for influenza complications in the outpatient setting, should receive antiviral treatment as soon as possible for suspected or confirmed influenza (196). Zambon and Haydon (89) emphasized the timely use of currently available antivirals during the threat of the influenza A(H3N2) Subclade K virus, for prophylaxis and treatment, to reduce the burden of this season’s anticipated epidemic. They further emphasized that chemoprophylaxis with influenza antivirals is a highly effective strategy for preventing illness and controlling outbreaks in both acute- and chronic-care facilities, as supported by multiple studies (89, 197–200).

There should also be a priority to fund research and development of improved antiviral drugs for therapeutic and prophylactic use in high-risk cohorts.

### 5.3 Monoclonal antibodies for influenza treatment or prevention

Therapeutic monoclonal antibodies offer distinct clinical and regulatory advantages over vaccines, particularly in the context of risk–benefit assessment. Unlike vaccines, which are administered broadly to healthy individuals for prophylaxis, monoclonal antibodies are typically given to patients who are already infected or at high risk of severe disease. This targeted use allows for a higher tolerance of potential adverse effects, as the anticipated therapeutic benefit outweighs the associated risks. In contrast, vaccines must demonstrate an exceptionally favorable safety profile, since they are administered to large, predominantly healthy populations with minimal immediate clinical benefit. Monoclonal antibody therapy also provides immediate passive immunity, delivering protection or treatment without the lag period required for an adaptive immune response to develop after vaccination. This is especially advantageous in immunocompromised or elderly individuals who may have a suboptimal response to vaccination. Moreover, therapeutic monoclonal antibodies can be precisely tailored to neutralize circulating viral strains, enabling rapid adaptation to antigenic drift and potentially achieving greater efficacy against emerging variants. Together, these attributes position therapeutic monoclonal antibodies as a complementary and, in certain contexts, superior intervention to vaccination, particularly for vulnerable or acutely infected populations where rapid, targeted, and risk-justified immune protection is required.

Passive immunotherapy, using hyperimmune sera or human immunoglobulins, has been employed to treat infectious diseases for over a century (201). As discussed above, during the 1918 influenza pandemic, administration of convalescent serum within four days of symptom onset reduced the absolute risk of death from 26% to 8%, demonstrating the protective role of HA-specific antibodies (12). Monoclonal antibodies are increasingly being used as treatment options, particularly for cancer and autoimmune diseases. More than 100 therapeutic biological drugs based on monoclonal antibodies have been approved (202) since the first approval of a monoclonal antibody product in 1986 (203). Crowe (42) proposed using combinations of monoclonal antibodies as influenza therapeutics or preventatives. However, challenges associated with using monoclonal antibodies during the annual influenza season for evolving new influenza strains include the cost and time required to generate them. Advances in recombinant technology, the development of Antibody Phage Display technology, and the delivery of monoclonal antibodies via mRNA have now made it possible to reduce the cost and time required to generate monoclonal antibodies for new strains of influenza viruses within 3–4 months (204). Since the development of Antibody Phage Display technology in the 1990s (205–207), almost more than 100 phage–derived antibodies have been evaluated in clinical studies, and approximately 19 of them have been approved (208–210).

Recent advances in mRNA-based delivery of monoclonal antibodies can further support efforts to deliver strain-specific seasonal influenza monoclonal antibodies. mRNA-encoded antibodies are an emerging therapeutic platform that enables rapid *in situ* production of potent neutralizing antibodies within the body (211, 212). Unlike conventional monoclonal antibody therapies, which rely on labor-intensive, time-consuming cell-culture manufacturing, mRNA approaches are faster, more easily scalable, and cell-free, eliminating the need for protein purification (212). Further work is ongoing to develop mRNA as a means of delivering monoclonal antibodies against infectious diseases, including influenza (213, 214). This could be an alternative to the need to purify monoclonal antibodies for formulation and delivery to people via intravenous or intramuscular injection.

Therapeutic monoclonal antibodies for seasonal influenza strains can be developed more rapidly for emerging strains by adopting a framework analogous to that of the annual influenza vaccine, and under the Platform Technology Designation Program of the 2022 “Prepare for and Respond to Existing Viruses, Emerging New Threats”, PREVENT Pandemics Act, Section 506K of the Federal Food, Drugs & Cosmetics Act (215, 216). This legal framework enables the 12-week technical timeline to deliver a distributed product by streamlining the review of “swapped” mRNA or mAb sequences (215, 216). In its Guidance, FDA has listed lipid nanoparticle (LNP) platforms for mRNA vaccine, gene therapy, or other products, and monoclonal antibody platform technologies that use the same cell substrate, expression construct, upstream and downstream processing (216). This approach involves submitting a Biologics License Application (BLA) for an LNP incorporating specific monoclonal antibodies mRNA product or purified monoclonal antibodies product, and obtaining approval, supported by comprehensive data demonstrating its safety and efficacy in humans. Once the manufacturing process for such a product has been approved under the initial BLA, subsequent approvals for antibodies targeting newly emergent strains may be pursued through BLA supplement filings, in a manner comparable to the annual update of influenza vaccines.

**Figure 6:**
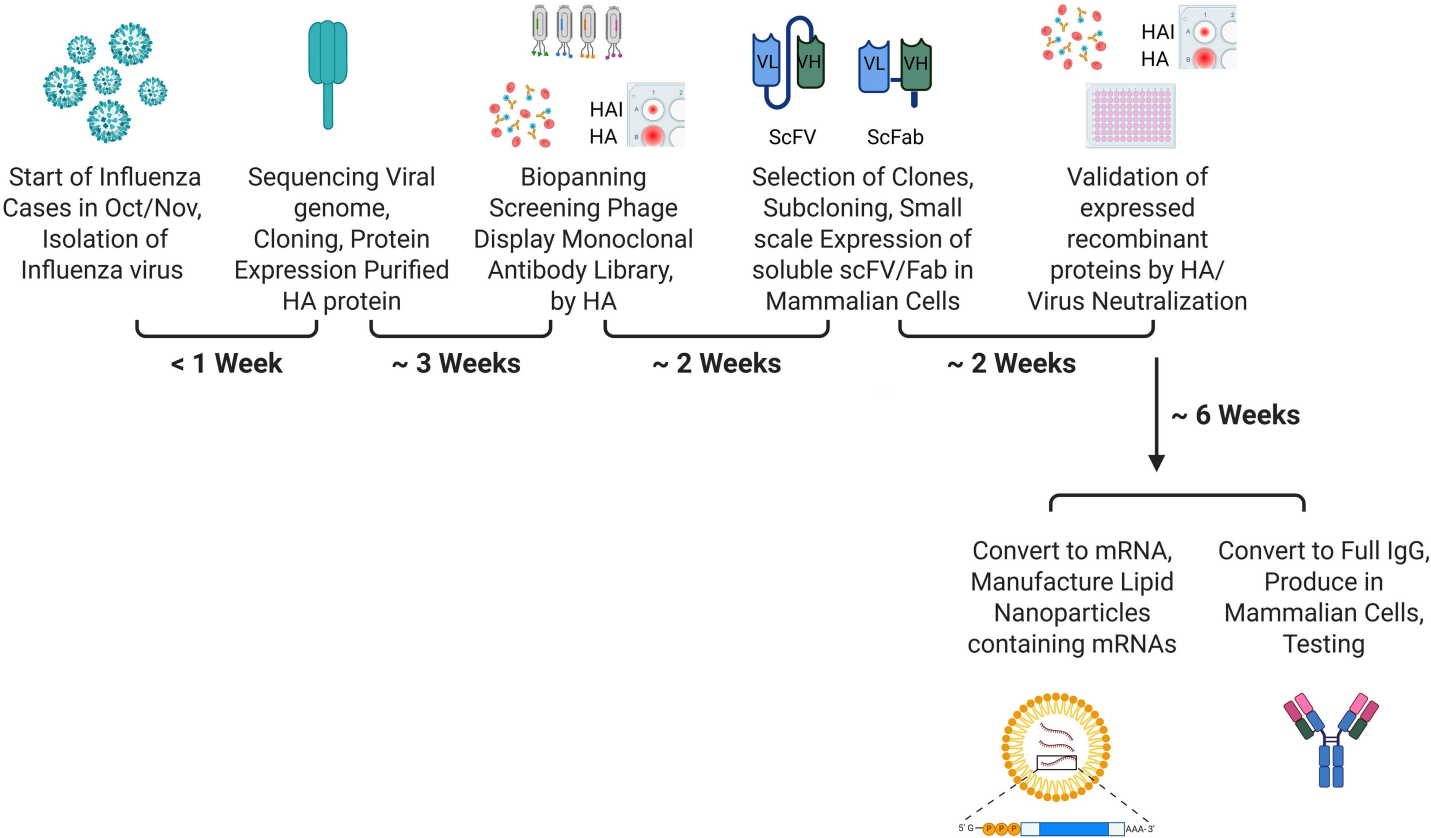
Development and manufacturing process for therapeutic monoclonal antibodies, or lipid nanoparticles containing specific monoclonal antibody mRNA, with timelines for controlling influenza after the emergence of new influenza strains. HAI - Hemagglutination Inhibition, HA Hemagglutination, scFV/Fab - Single chain variable (FV) and antigen-binding (ab) fractions. Gupta, R. (2026) https://BioRender.com/0dvtbzm.

**Figure 6** presents an overview of the process for generating recombinant monoclonal antibodies or LNP-mRNA for a specific monoclonal antibody, based on the HA epitope structure of a novel influenza strain. When new influenza strains are isolated from patients in October/November, the viral genome will be sequenced, and appropriate epitopes from the HA spike protein will be designed, including the use of an in-silico epitope mapping and artificial intelligence to screen HA structures and rapidly identify potential epitopes for protective antibodies. Selected peptides with conformational epitopes will be manufactured for bio-panning using a commercial synthetic antibody phage display library or a naïve antibody phage display library. Strain-specific HA monoclonal antibodies or LNP-mRNA for a specific monoclonal antibody can be generated in approximately 3–4 months, representing a substantial reduction in development time relative to the six or more months typically required for egg-based vaccine production for predicted, rather than circulating strains. Tan et al. (204) consistently implemented an innovative and accelerated cell line development (CLD) workflow and an expedited chemistry, manufacturing, and control (CMC) strategy to develop multiple SARS‐CoV‐2‐neutralizing monoclonal antibody programs. This resulted in greatly reduced CMC timelines from DNA to Investigational New Drug (IND) for these monoclonal antibodies. This strategy enabled them to have clinical materials ready in as little as 3 months to address the urgent, unmet medical needs during the COVID‐19 pandemic. The authors attributed the successful execution of this expedited CMC strategy to a proven and robust platform process, especially CLD, and effective collaboration among different functions within the CMC areas (216).

New influenza strains generally appear in October or November (57, 58). The A/H3N2 subclade K strain, which caused most influenza cases during the 2025–26 season, was the dominant circulating strain in many countries by May and November 2025, and was reported by the CDC in August 2025 (89, 92). By leveraging global surveillance data on circulating strains, monoclonal antibodies against these variants could be developed by the end of January, around the time influenza cases begin to peak. Until monoclonal antibodies are manufactured, influenza cases should be treated with antivirals, as discussed above. When monoclonal antibodies are ready for use, they can be used for both the treatment of influenza and prophylaxis in vulnerable populations, such as the elderly and infants. The potential development and effective prophylactic use of monoclonal antibodies against HA are supported by the successful development of long-acting monoclonal antibodies to the F protein of RSV (217–219). These antibodies can be administered via intramuscular injection and have an extended half-life, providing protection for up to 5 months. Development can be expedited by using Southern Hemisphere data from global surveillance of circulating influenza strains to start cell line development or mRNA design in June. This proactive approach ensures that once specific influenza strains emerge in October/November, the manufacture of targeted monoclonal antibodies or LNP-mRNA can begin immediately.

Recently published data suggested that an alternative would be to develop a monoclonal cocktail targeting HA head regions, conserved stalk HA regions, and conserved regions of the NA (220). Hoy et al. (221) found that household contacts with high levels of preexisting antibodies to the HA head and stalk and to NA had reduced susceptibility to influenza infection. Momont et al. (222) reported the development of a monoclonal antibody to NA that exhibited viral-neutralizing activity against a wide variety of influenza strains. Lei et al. (223) also reported three NA antibodies conferring prophylactic and therapeutic protection *in vivo*, mediated by both Fc effector functions and NA inhibition via steric hindrance in mice. Clearly, the development of effective, broadly neutralizing antibodies that could be delivered via intramuscular injection and have an extended half-life would be ideal.

### 5.4 Influenza active vaccination strategy and new vaccines

As discussed above, annual influenza vaccination has low and variable effectiveness, not only because of antigenic drift that can result in an HA antigenic mismatch with circulating clinical strains, but also because it can stimulate immune tolerance, which can blunt the production of functional antibodies to HA. Influenza vaccines are not very effective, particularly at providing infection-blocking or sterilizing immunity (preventing infection and transmission) with diminishing immunological returns due to antigenic drift and repeated annual vaccination. From an immunodynamic perspective, infection-blocking immunity (antibody titers) is often immunologically attenuating or wanes over time: antigenic drift can erode epitope matching, leading to a decline in sterilizing protection. Repeated antigenic exposures (via infection or vaccination) may transiently restore this barrier, but gains are frequently subject to diminishing returns, as successive boosts preferentially expand pre-existing memory B-cell clones targeting conserved epitopes rather than generating robust *de novo* responses to novel antigenic sites (a pattern consistent with immune imprinting frameworks).

It is proposed that the annual influenza vaccination should be reevaluated. New vaccines comprising HA from potential circulating strains should be developed using recombinant protein expression systems and mRNA vaccine technology rather than animal cell culture or egg-based production. In addition, the targeted vaccine HA antigens should be selected from viral sequences obtained directly from clinical isolates, before passage in eggs or in cell cultures, as these passages have been shown to alter the RBD of the HA antigen (discussed in sections 4.3 and 4.4).

Vaccines that confer very long-term or lifelong protection are primarily live attenuated vaccines, such as smallpox, MMR, chickenpox, polio, and yellow fever. Antibody responses following viral infections or live vaccines have half-lives of 50 years or more (48). This could also be because the protection is primarily mediated by cytotoxic CD8^+^ and CD4 cells specific to conserved antigens. A live-attenuated flu vaccine, FluMist, has been approved since 2003 (224). However, highly variable effectiveness across seasons, including markedly reduced performance of the A(H1N1)pdm09 component after 2009, highlighted fundamental challenges in live attenuated influenza vaccine design, such as restricted replicative fitness of certain reassortants, interference among multivalent components, and the difficulty of achieving balanced, stable attenuation while preserving robust immunogenicity. Further, FluMist is grown in eggs and thus is subject to HA antigenic changes from circulating wild-type strains, as discussed above in section 4.3. Other options for a better live influenza vaccine need to be explored.

The licensure of mRNA vaccines against SARS-CoV-2 in 2020–2021 fundamentally altered the trajectory of vaccine development. The unprecedented efficacy and rapid scalability of mRNA vaccines, built on decades of work in nucleoside-modified mRNA and lipid nanoparticle delivery, provided the first global demonstration that mRNA vaccines can elicit safe, real-time responses to emerging viral threats (225, 226). This success has naturally accelerated interest in applying the platform to influenza (125–128, 227, 228). Recent clinical studies of seasonal influenza mRNA candidates show strong immunogenicity, greater efficacy than current licensed egg-based vaccines, and acceptable reactogenicity (125–128), reinforcing the platform’s potential public -health value (125). Advantages of mRNA-based influenza vaccines over current vaccines include HA antigens that match the sequences of circulating influenza viruses, faster antigen updates, higher sequence fidelity, rapid manufacturing scale-up, and the possibility of multivalent inclusion of several hemagglutinin antigens or combined influenza–COVID-19 constructs, broadening immunogenic potential beyond what quadrivalent inactivated vaccines can deliver. Instead of developing mRNA vaccines from the strains selected in February (discussed in Section 4.2), these vaccines, like monoclonal antibodies (discussed in Section 5.3), can be developed from strains that begin circulating in October or November or even earlier for use during peak influenza season, starting in January/February of the following year. As discussed, the sequence of the A/H3N2 subclade K strain, which caused most influenza cases during the 2025–26 season (89–91), was available at the CDC in August 2025 (92). The mRNA-based vaccine for this new subclade could have been developed by the end of the year for use during the peak of the 2025–26 influenza season. However, these advantages of mRNA vaccines are subject to diminishing returns due to constraints imposed by immune imprinting via OAS, or antigenic superiority, and antigenic drift.

The more consequential issue with the mRNA vaccines lies in the regulatory interface with the FDA’s Center for Biologics Evaluation and Research (CBER). In 2026, the FDA issued a rare Refusal-to-File (RTF) for Moderna’s Biologics License Application (BLA) for mRNA-1010, not on the basis of safety or efficacy concerns, but due to clinical trial design, specifically, the choice of comparator vaccine. The agency argued that the use of a standard-dose comparator in older adults failed to reflect the current standard of care (i.e., high-dose or adjuvanted vaccines), thereby undermining the interpretability of relative efficacy claims in the most clinically relevant population. This is a non-trivial regulatory signal: for influenza vaccines, licensure remains anchored in comparative effectiveness against licensed products rather than immunobridging alone, and comparator selection is now a critical determinant of BLA acceptability. The CBER quickly relented and filed the BLA. Regulatory agility must keep pace with technological speed and scientific advances to bring innovative products to patients in a timely manner.

Other approaches could include further development of purified, recombinantly expressed HA and NA proteins from viral sequences obtained directly from clinical isolates, using appropriate host systems, such as insect cell culture, which has been successfully used for different HAs in the licensed Flublok vaccine. Recombinantly expressed chimeric proteins from conserved epitopes in the stem and head regions of HA and NA could also be used to develop alternate vaccines. For more than 50 years, it has been known that antibodies to NA protect against infection during seasonal and pandemic influenza outbreaks (191). The inclusion of NA antigens in potentially improved influenza vaccines is also supported by recent findings (221–223) that monoclonal antibodies to conserved NA epitopes exhibit cross-reactive viral-neutralizing activity and from preclinical animal model data on the use of a purified recombinant NA antigen. Vaccines containing optimal levels of NA may be particularly useful when there is antigenic drift or shift in the HA because NA immunity offers broad protection (191).

An adjuvant that can help elicit memory B cells (229–232) may improve the level and duration of the immune response with a lower antigen dose with purified recombinant proteins, including chimeric proteins, as influenza vaccines appear to elicit a weak B cell memory response compared to the strong response observed after influenza infection. Many types of adjuvants have been evaluated for inclusion in various influenza vaccine formulations (233). However, the only annual influenza vaccine that contains an adjuvant is Fluad, which is licensed for use in individuals 65 years and older and contains MF59, an oil-in-water emulsion (233). While this adjuvant can increase HAI antibody titers over non-adjuvanted vaccines (233) and can provide improved protection over standard non-adjuvanted vaccines in the elderly, the adjuvanted and non-adjuvanted high-dose Fluzone vaccines were very similar in the level of protection against symptomatic disease (233, 234). An adjuvant that is now receiving considerable attention for inclusion in both purified subunit and mRNA influenza vaccines is the CpG oligonucleotide. This adjuvant enhances the immune response in the licensed Hepatitis B subunit vaccine, HEPLISAV B (235), and in preclinical animal models has enhanced the memory B-cell and T-cell responses to a multivalent mRNA formulation expressing different influenza HA antigens (236) and to a purified recombinant influenza NA antigen (237). CpG may have additional properties compared with other adjuvants for influenza vaccines, as suggested by Kakh et al. (39), because it could reduce the stimulation and expansion of antigen-specific T-reg cells, thereby enhancing vaccine effectiveness.

Newly developed recombinant protein and mRNA influenza vaccines that elicit memory B cells should be evaluated as childhood vaccines for primary immunization, particularly in young children without a history of annual influenza vaccination, to avoid non-response due to OAS or antigenic superiority and immune tolerance. As discussed in Section 4.5, there are numerous scientific reports on the reduction in influenza vaccine effectiveness after repeated vaccinations (49, 120–122, 129–159). Also, influenza vaccination effectively reduced life-threatening influenza illness by 75% in critically ill children with acute respiratory infection during a season predominated by B/Victoria viruses and A/H1N1pdm09 subclade viruses that were antigenically drifted from vaccine components (238), when low protection was observed in adults during the same season (239). Olson et al. (238) speculated that immune responses and correlates of protection in children, who are more likely to be immunologically naïve, differ from those in adults, who are more likely to be boosted by repeated infections and vaccinations (240). Children are important vectors of influenza in the community due to their movement between schools and households (237, 241–244), and due to high viral loads that can extend the viral shedding period for transmission of the virus to adult teachers, parents, and grandparents. Tanner et al. (87) also suggested vaccinating children as a possible way to protect against influenza.

## 6 Improvement of the effectiveness of the influenza vaccine on broad public health impact

Improving the effectiveness of influenza vaccines could not only enhance influenza control but also strengthen control of other respiratory pathogens by boosting public trust in other vaccines. The low and variable year-to-year effectiveness of influenza vaccines (**Figure 1**) presents a challenge to vaccine uptake, contributing to vaccine hesitancy, as individuals question the benefits and necessity of vaccination when perceived protection is limited. When the public hears that the influenza vaccine’s effectiveness was lower in a particular previous influenza season and in the current season (245), it leads to a perception that the vaccine “doesn’t work.” This can be particularly damaging when individuals and their peers who received the vaccine still get the disease, leading to a sense of betrayal or futility. In an era of increased vaccination efforts (e.g., COVID-19), “vaccine fatigue” can set in (246). If the influenza vaccine is seen as less effective, it might be the first one people decide to skip.

Low effectiveness compounds existing fears about vaccine safety, leading to skepticism about pharmaceutical companies and public health recommendations, and fueling a lack of trust in health authorities or the necessity of annual vaccines, impacting attitudes toward influenza vaccination and potentially vaccines in general. Studies have shown that perceptions of low vaccine effectiveness correlate strongly with vaccine hesitancy and reduced uptake (247–249).

Further, when vaccine effectiveness is low, public messaging about the residual benefits (such as reduction in severe disease or hospitalizations) is often insufficient to overcome reluctance, especially among those with limited trust in authorities or limited knowledge of how vaccines work. In the 2024–2025 season, evidence suggesting that the vaccine may have offered negligible or even negative effectiveness in some populations may exacerbate this effect (250). Healthcare workers, who are crucial in recommending vaccination, may also experience reduced motivation to promote the annual influenza vaccine if they perceive its effectiveness to be low.

Improving the effectiveness of influenza vaccines could also be important by diminishing the impact of reduced use of preventive behavioral measures due to vaccination complacency. Preventive behavioral measures during the winter months, such as mask-wearing, handwashing, and social distancing or contact avoidance, play a crucial role in controlling several seasonal respiratory infections and diseases, including influenza, COVID-19, the common cold, respiratory syncytial virus infections, and emerging human metapneumovirus (hMPV) infections. Vaccinated individuals, particularly those against influenza with uncertain effectiveness, negatively influence preventive behavior among vaccinees due to a perceived lower risk of infection (251). Preventive behavioral measures, particularly contact avoidance, significantly reduce disease transmission (252–254), and the perceived risk of infection is essential in practicing such behavioral responses (249). In general, it has been observed that vaccinated individuals do not engage in avoidance behavior to the same extent as unvaccinated individuals, which may influence their level of engagement in such behavior compared with unvaccinated individuals (250). Vilches et al. (255) demonstrated that vaccination can lead to a larger epidemic size if the avoidance level of vaccinated individuals is below that of susceptible individuals. They also emphasized that seasonal influenza immunization programs should enhance strategies to promote preventive behavioral measures. Improved influenza vaccines that are more effective at preventing infection and/or transmission of the virus in vaccinated individuals would help to mitigate the effects of vaccination complacency.

## 7 Conclusion

While most vaccines confer long-lasting protection through memory responses, annual influenza vaccination struggles with low and variable effectiveness. This is attributed to factors such as antigenic drift, mismatches between egg-adapted or cell-culture passaged vaccine strains and circulating wild-type strains, repeated vaccination, and the phenomenon of original antigenic sin. Repeated annual influenza vaccination has been associated with a “blunting” of the antibody response, particularly to new antigens on emerging strains. The antibody blunting may be driven by the expansion and enhanced regulatory activity of Tfr cells and/or antigenic seniority or the OAS phenomenon. Contrary to the typical vaccine paradigm, we argue that the current annual influenza vaccination policy is not very effective due to the blunting of the production of viral neutralizing antibodies to HA upon repeated immunization with the same antigenic HA structures. A paradigm shift is needed, moving towards alternative control strategies (improved antivirals, monoclonal antibodies, and behavioral measures) alongside the development of vaccines based on recombinant proteins and mRNA technology. Improved vaccine designs can include formulations containing key variable and conserved epitopes, along with adjuvants to modulate Tfr responses, thereby enhancing activation of influenza-specific B and T cells and promoting short-term and durable memory immunity. For example, future multivalent vaccines based on purified, recombinantly expressed proteins and mRNA vaccine technology that stimulate viral-neutralizing and ADCC antibodies against HA and NA, in addition to stimulating cytotoxic T-cells against conserved viral proteins expressed on the surface of infected cells, could be much more effective than currently used influenza vaccines. Immunological insights, discussed in this article, suggest that vaccine platforms and adjuvants should be optimized not only to enhance Tfh responses but also to transiently limit early Tfr dominance. Strategies that favor pro-Tfh cytokine environments (e.g., IL-6, IL-12) during priming, while allowing delayed Tfr-mediated contraction, may improve both the magnitude and durability of antibody responses. Conversely, formulations that induce early IL-10/TGF-β–rich environments risk attenuating GC initiation and thereby blunting protective immunity.

## References

[1] Rodrigues CMC Plotkin SA . Impact of vaccines; health, economic and social perspectives. Front Microbiol. (2020) 11:Article 1526. doi: 10.3389/fmicb.2020.01526. PMID: 32760367 PMC7371956

[2] World Health Organization . The Global Eradication of Smallpox: Final Report of the Global Commission for the Certification of Smallpox Eradication, Geneva, December 1979. Geneva, Switzerland: World Health Organization (1980). Available online at: https://iris.who.int/server/api/core/bitstreams/d98b8bb1-e8e4-4845-9879-04b373dc235c/content (Accessed April 29, 2026).

[3] Centers for Disease Control and Prevention . Certification of poliomyelitis eradication: the Americas. MMWR. (1994) 43:720–2. Available online at: https://www.cdc.gov/mmwr/preview/mmwrhtml/00032760.htm.

[4] Mark P Califf R . Is vaccination approaching a dangerous tipping point? JAMA. (2024) 331:283–4. doi: 10.1001/jama.2023.27685. PMID: 38180773

[5] Gupta RK . Hepatitis B vaccination at birth: science, myths, and the safety net imperative. Front Immunol. (2026) 17:1770223. doi: 10.3389/fimmu.2026.1770223. PMID: 41924273 PMC13036139

[6] Centers for Disease Control and Prevention . Prevention and control of influenza with vaccines. Recommendations of the Advisory Committee on Immunization Practices (ACIP), 2010. MMWR. (2010) 59:1–62. doi: 10.1542/9781610020862-part06-prevention_and 20689501

[7] Belongia EA Simpson MD King JP Sundaram ME Kelley NS Osterholm MT . Variable influenza vaccine effectiveness by subtype: a systematic review and meta-analysis of test-negative design studies. Lancet Infect Dis. (2016) 6:942–51. doi: 10.1016/S1473-3099(16)00129-8. PMID: 27061888

[8] Nicoll A Sprenger M . Low effectiveness undermines promotion of seasonal influenza vaccine. Lancet Infect Dis. (2013) 13:7–9. doi: 10.1016/S1473-3099(12)70313-4. PMID: 23257219

[9] Flannery B Chung JR Belongia EA McLean HQ Gaglani M Murthy K . Interim estimates of 2017–18 seasonal influenza vaccine effectiveness — United States, February 2018. MMWR. (2018) 67:180–5. doi: 10.15585/mmwr.mm6706a2. PMID: 29447141 PMC5815489

[10] Paules CI Sullivan SG Subbarao K Fauci AS . Chasing seasonal influenza - the need for a universal influenza vaccine. N Engl J Med. (2018) 378:7–9. doi: 10.1056/NEJMp1714916. PMID: 29185857

[11] Skowronski DM Chambers C De Serres G Dickinson JA Winter AL Hickman R . Early season co-circulation of influenza A(H3N2) and B(Yamagata): interim estimates of 2017/18 vaccine effectiveness, Canada, January 2018. Euro Surveill. (2018) 23:18–00035. doi: 10.2807/1560-7917.ES.2018.23.5.18-00035. PMID: 29409570 PMC5801641

[12] Bresee JS Fry AM Sambhara S Cox NJ . In Plotkin's vaccines. Plotkin SA Orenstein WA Paul A Offit PA Edwards KM , editors. Elsevier (2018). p. 456–488e18. doi: 10.1016/B978-0-323-35761-6.00031-6

[13] Gaglani M Vasudevan A Raiyani C Murthy K Chen W Reia M . Effectiveness of trivalent and quadrivalent inactivated vaccines against influenza B in the United States, 2011–2012 to 2016-2017. Clin Infect Dis. (2021) 72:1147–57. doi: 10.1093/cid/ciaa102. PMID: 32006430 PMC8028105

[14] Ambrose CS Levin MJ . The rationale for quadrivalent influenza vaccines. Hum Vaccin Immunother. (2012) 8:81–8. doi: 10.4161/hv.8.1.17623. PMID: 22252006 PMC3350141

[15] Koutsakos M Wheatley AK Laurie K Kent SJ Rockman S . Influenza lineage extinction during the COVID-19 pandemic? Nat Rev Microbiol. (2021) 19:741–2. doi: 10.1038/s41579-021-00642-4. PMID: 34584246 PMC8477979

[16] Caini S Meijer A Nunes MC Henaff L Zounon M Boudesijns B . Probable extinction of influenza B/Yamagata and its public health implications: a systematic literature review and assessment of global surveillance databases. Lancet Microbe. (2024) 5:100851. doi: 10.1016/S2666-5247(24)00066-1. PMID: 38729197

[17] Food and Drug Administration . Use of Trivalent Influenza Vaccines for the 2024–2025 U.S. Influenza Season. Available online at: https://www.fda.gov/vaccines-blood-biologics/lot-release/use-trivalent-influenza-vaccines-2024-2025-us-influenza-season#:~:text=The%20FDA%20has%20been%20working%20with%20manufacturers,A/Wisconsin/67/2022%20(H1N1)pdm09%2Dlike%20virus%20&ast;%20A/Massachusetts/18/2022%20(H3N2)%2Dlike%20virus (Accessed April 29, 2026).

[18] Agor JK Özaltın OY . Models for predicting the evolution of influenza to inform vaccine strain selection. Hum Vaccines Immunother. (2018) 14:678–83. doi: 10.1080/21645515.2017.1423152. PMID: 29337643 PMC5861780

[19] de Rooij AJH Lempers VJC Park Y Vicic N Han AX Russell CA . Reproducible and later vaccine strain selection can improve vaccine match to A/H3N2 seasonal influenza viruses. NPJ Vaccines. (2025) 10:243. doi: 10.1038/s41541-025-01292-w. PMID: 41271798 PMC12638742

[20] Centers for Disease Control and Prevention . Selecting Viruses for the Seasonal Influenza Vaccine. Available online at: https://www.cdc.gov/flu/vaccine-process/vaccine-selection.html (Accessed April 29, 2026).

[21] Centers for Disease Control and Prevention . Seasonal Flu Vaccine Effectiveness Studies. Available online at: https://www.cdc.gov/flu-vaccines-work/php/effectiveness-studies/index.html (Accessed April 29, 2026).

[22] Centers for Disease Control and Prevention . Flu Vaccination Coverage, United States, 2023–24 Influenza Season. Available online at: https://www.cdc.gov/fluvaxview/coverage-by-season/2023-2024.html#:~:text=In%20the%202023%E2%80%9324%20flu,%E2%80%9320%20season%20(63.7%25) (Accessed April 29, 2026).

[23] Robbins JB Schneerson R Szu SC . Perspective: hypothesis: serum IgG antibody is sufficient to confer protection against infectious diseases by inactivating the inoculum. J Infect Dis. (1995) 171:1387–98. doi: 10.1093/infdis/171.6.1387. PMID: 7769272

[24] Siegrist C-A . Vaccine immunology. In: Plotkin's Vaccines Plotkin SA Orenstein WA Paul A Offit PA Edwards KM , editors. Philadelphia, PA: Elsevier (2018). p. 16–34.e7. doi: 10.1016/B978-0-323-35761-6.00002-X

[25] Song Y Mehl F Zeichner SL . Vaccine strategies to elicit mucosal immunity. Vaccines. (2024) 12:191. doi: 10.3390/vaccines12020191. PMID: 38400174 PMC10892965

[26] Alqahtani SAM . Mucosal immunity in COVID-19: a comprehensive review. Front Immunol. (2024) 15:1433452. doi: 10.3389/fimmu.2024.1433452. PMID: 39206184 PMC11349522

[27] Rubin R . In search of COVID-19 vaccines that elicit mucosal immunity and stop transmission. JAMA. (2025) 333:187–9. doi: 10.1001/jama.2024.23627. PMID: 39705023

[28] MacLennan IC Toellner KM Cunningham AF Serre K Sze DM Zúñiga E . Extrafollicular antibody responses. Immunol Rev. (2003) 194:8–18. doi: 10.1034/j.1600-065x.2003.00058.x. PMID: 12846803

[29] Goodnow CC Vinuesa CG Randall KL Mackay F Brink R . Control systems and decision making for antibody production. Nat Immunol. (2010) 11:681–8. doi: 10.1038/ni.1900. PMID: 20644574

[30] Palucka K Banchereau J Mellman I . Designing vaccines based on biology of human dendritic cell subsets. Immunity. (2010) 33:464–78. doi: 10.1016/j.immuni.2010.10.007. PMID: 21029958 PMC2975953

[31] Cyster JG Allen CDC . B cell responses: cell interaction dynamics and decisions. Cell. (2019) 177:524–40. doi: 10.1016/j.cell.2019.03.016. PMID: 31002794 PMC6538279

[32] Beissert S Schwarz A Schwarz T . Regulatory T cells. J Invest Dermat. (2006) 126:15–24. doi: 10.1038/sj.jid.5700004. PMID: 16417213

[33] Sage PT Sharpe AH . T follicular regulatory cells in the regulation of B cell responses. Trends Immunol. (2015) 36:410–8. doi: 10.1016/j.it.2015.05.005. PMID: 26091728 PMC4508020

[34] Plitas G Rudensky AY . Regulatory T cells: differentiation and function. Cancer Immunol Res. (2016) 4:721–5. doi: 10.1158/2326-6066.CIR-16-0193. PMID: 27590281 PMC5026325

[35] Xie MM Dent AL . Unexpected help: follicular regulatory T cells in the germinal center. Front Immunol. (2018) 9:1536. doi: 10.3389/fimmu.2018.01536. PMID: 30013575 PMC6036241

[36] Kurosaki T Kometani K Wataru I . Memory B cells. Nat Rev Immunol. (2015) 15:149–59. doi: 10.1038/nri3802. PMID: 25677494

[37] Krogsgaard M Davis MM . How T cells “see” antigen. Nat Immunol. (2005) 6:239–45. doi: 10.1038/ni1173. PMID: 15716973

[38] Sjaastad LE Owen DL Tracy SI Farrar MA . Phenotypic and functional diversity in regulatory T cells. Front Cell Dev Biol. (2021) 9:715901. doi: 10.3389/fcell.2021.715901. PMID: 34631704 PMC8495164

[39] Kakh MK Doroudchi M Talepoor A . Induction of regulatory T cells after virus infection and vaccination. Immunology. (2025), 1–16. doi: 10.1111/imm.13927. PMID: 40329764

[40] Gong Q Sharma M Glass MC Kuan EL Chander A Singh M . Multi-omic profiling reveals age-related immune dynamics in healthy adults. Nature. (2025) 648:696–706. doi: 10.1038/s41586-025-09686-5. PMID: 41162704 PMC12711581

[41] Rudman Spergel AK Lee IT Koslovsky K Schaefers K Avanesov A Logan DK . Immunogenicity and safety of mRNA-based seasonal influenza vaccines encoding hemagglutinin and neuraminidase. Nat Commun. (2025) 16:5933. doi: 10.1038/s41467-025-60938-4. PMID: 40595624 PMC12216828

[42] Crowe JE . Antibody determinants of influenza immunity. J Infect Dis. (2019) 219:S21–9. doi: 10.1093/infdis/jiz010. PMID: 30715373 PMC6452307

[43] Kosikova M Li L Radvak P Ye Z Wan XF Xie H . Imprinting of repeated influenza A/H3 exposures on antibody quantity and antibody quality: implications for seasonal vaccine strain selection and vaccine performance. Clin Infect Dis. (2018) 67:1523–32. doi: 10.1093/cid/ciy327. PMID: 29672713 PMC6206119

[44] Verschoor CP Singh P Russell ML Bowdish DME Brewer A Cyr L . Microneutralization assay titres correlate with protection against seasonal influenza H1N1 and H3N2 in children. PloS One. (2015) 10:e0131531. doi: 10.1371/journal.pone.0131531. PMID: 26107625 PMC4479562

[45] Paccalin M Plouzeau C Bouche G Guillard O Beby-Defaux A Mauco G . Lack of correlation between nutritional status and seroprotection against influenza in a long term care facility. Scand J Infect Dis. (2006) 38:894–7. doi: 10.1080/00365540600749984. PMID: 17008234

[46] Vajo Z Laszlofy C . Developing correlates of protection for vaccines is needed more than ever-influenza, COVID-19 and RSV infection. Viruses. (2024) 16:1671. doi: 10.3390/v16111671. PMID: 39599786 PMC11598905

[47] Houser K Subbarao K . Influenza vaccines: challenges and solutions. Cell Host Microbe. (2015) 17:295–300. doi: 10.1016/j.chom.2015.02.012. PMID: 25766291 PMC4362519

[48] Amanna IJ Carlson NE Slifka MK . Duration of humoral immunity to common viral and vaccine antigens. N Engl J Med. (2007) 357:1903–15. doi: 10.1056/NEJMoa066092. PMID: 17989383

[49] Poland GA . Influenza vaccine failure: failure to protect or failure to understand? Expert Rev Vaccines. (2018) 17:495–502. doi: 10.1080/14760584.2018.1484284. PMID: 29883218 PMC6330882

[50] Cox RJ Haaheim LR Ericsson JC Madhun AS Brokstad KA . The humoral and cellular responses induced locally and systemically after parenteral influenza vaccination in man. Vaccine. (2006) 24:6577–80. doi: 10.1016/j.vaccine.2006.05.041. PMID: 16842889

[51] Jacobson RM Grill DE Oberg AL Tosh PK Ovsyannikova IG Poland GA . Profiles of influenza A/H1N1 vaccine response using hemagglutination-inhibition titers. Hum Vaccine Immunother. (2015) 11:961–9. doi: 10.1080/21645515.2015.1011990. PMID: 25835513 PMC4514374

[52] Young B Zhao X Cook AR Parry CM Wilder-Smith A I-cheng MC . Do antibody responses to the influenza vaccine persist year-round in the elderly? A systematic review and meta-analysis. Vaccine. (2017) 35:212–21. doi: 10.1016/j.vaccine.2016.11.013. PMID: 27939013

[53] European Centre for Disease Prevention and Control . WHO recommendations for influenza virus vaccine composition for the 2017 southern hemisphere season, 10 Oct 2016. Available online at: https://www.ecdc.europa.eu/en/news-events/who-recommendations-influenza-virus-vaccine-composition-2017-southern-hemisphere-season (Accessed April 29, 2026).

[54] CIDRAP, University of Minnesota . WHO changes H1N1 flu vaccine strain, punts on H3N2 (2019). Available online at: https://www.cidrap.umn.edu/influenza-vaccines/who-changes-h1n1-flu-vaccine-strain-punts-h3n2 (Accessed April 29, 2026).

[55] Global Influenza Surveillance and Response System, GISAID . Human Influenza Vaccine Composition. Available online at: https://gisaid.org/resources/human-influenza-vaccine-composition/ (Accessed April 29, 2026).

[56] Friedman N Drori Y Pando R Glatman-Freedman A Sefty H Bassal R . A(H1N1)pdm09 influenza infection: vaccine inefficiency. Oncotarget. (2017) 8:32856–63. doi: 10.18632/oncotarget.16459. PMID: 28415629 PMC5464833

[57] Centers for Disease Control and Prevention . Influenza Activity in the United States during the 2023–2024 Season and Composition of the 2024–2025 Influenza Vaccine (2024). Available online at: https://www.cdc.gov/flu/whats-new/flu-summary-2023-2024.html (Accessed April 29, 2026).

[58] Centers for Disease Control and Prevention . Influenza Activity in the United States during the 2024–25 Season and Composition of the 2025–26 Influenza Vaccine (2025). Available online at: https://www.cdc.gov/flu/whats-new/2025-2026-influenza-activity.html (Accessed April 29, 2026).

[59] Krause JC Tumpey TM Huffman CJ McGraw PA Pearce MB Tsibane T . Naturally occurring human monoclonal antibodies neutralize both 1918 and 2009 pandemic influenza A (H1N1) viruses. J Virol. (2010) 84:3127–30. doi: 10.1128/JVI.02184-09. PMID: 20042511 PMC2826039

[60] Yu X Tsibane T McGraw PA House FS Keefer CJ Hicar MD . Neutralizing antibodies derived from the B cells of 1918 influenza pandemic survivors. Nature. (2008) 455:532–6. doi: 10.1038/nature07231. PMID: 18716625 PMC2848880

[61] Xu R Ekiert DC Krause JC Hai R Crowe JE Wilson IA . Structural basis of preexisting immunity to the 2009 H1N1 pandemic influenza virus. Science. (2010) 328:357–60. doi: 10.1126/science.1186430. PMID: 20339031 PMC2897825

[62] Tsibane T Ekiert DC Krause JC Martinez O Crowe Jr JE Wilson I . Influenza human monoclonal antibody 1F1 interacts with three major antigenic sites and residues mediating human receptor specificity in H1N1 viruses. PloS Pathog. (2012) 8:e1003067. doi: 10.1371/journal.ppat.1003067. PMID: 23236279 PMC3516549

[63] Li Y Myers JL Bostick DL Sullivan CB Madara J Linderman SL . Immune history shapes specificity of pandemic H1N1 influenza antibody responses. J Exp Med. (2013) 210:1493–500. doi: 10.1084/jem.20130212. PMID: 23857983 PMC3727314

[64] Linderman SL Chambers BS Zost SJ Parkhouse K Li Y Herrmann C . Potential antigenic explanation for atypical H1N1 infections among middle-aged adults during the 2013–2014 influenza season. Proc Natl Acad Sci. (2014) 111:15798–803. doi: 10.1073/pnas.1409171111. PMID: 25331901 PMC4226110

[65] Cobey S Hensley SE . Immune history and influenza virus susceptibility. Curr Opin Virol. (2017) 22:105–11. doi: 10.1016/j.coviro.2016.12.004. PMID: 28088686 PMC5467731

[66] McElhaney JE Ewen C Zhou X Kane KP Xie D Hager WD . Granzyme B: Correlates with protection and enhanced CTL response to influenza vaccination in older adults. Vaccine. (2009) 27:2418–25. doi: 10.1016/j.vaccine.2009.01.136. PMID: 19368783 PMC2800816

[67] Müllbacher A Lobigs M Alsharifi M Regner M . Cytotoxic T-cell immunity as a target for influenza vaccines. Lancet Infect Dis. (2006) 6:255–6. doi: 10.1016/S1473-3099(06)70443-1. PMID: 16631540

[68] Corti D Voss J Gamblin SJ Codoni G Macagno A Jarrossay D . A neutralizing antibody selected from plasma cells that binds to group 1 and group 2 influenza A hemagglutinins. Science. (2011) 333:850–6. doi: 10.1126/science.1205669. PMID: 21798894

[69] Leon PE He W Mullarkey CE Tan GS . Optimal activation of Fc-mediated effector functions by influenza virus hemagglutinin antibodies requires two points of contact. Proc Natl Acad Sci. (2016) 113:E5944–51. doi: 10.1073/pnas.1613225113. PMID: 27647907 PMC5056099

[70] Wang Q Tian X Chen X Ma J . Structural basis for receptor specificity of influenza B virus hemagglutinin. Proc Natl Acad Sci. (2007) 104:16874–9. doi: 10.1073/pnas.0708363104. PMID: 17942670 PMC2040455

[71] Chung Y Tanaka S Chu F Nurieva RI Martinez GJ Rawal S . Follicular regulatory T cells expressing Foxp3 and Bcl-6 suppress germinal center reactions. Nat Med. (2011) 17:983–8. doi: 10.1038/nm.2426. PMID: 21785430 PMC3151340

[72] Linterman MA Pierson W Lee SK Kallies A Kawamoto S Rayner TF . Foxp3+ follicular regulatory T cells control the germinal center response. Nat Med. (2011) 17:975–82. doi: 10.1038/nm.2425. PMID: 21785433 PMC3182542

[73] Wing JB Kitagawa Y Locci M Hume H Tay C Morita T . A distinct subpopulation of CD25- T-follicular regulatory cells localizes in the germinal centers. Proc Natl Acad Sci USA. (2017) 114:E6400–9. doi: 10.1073/pnas.1705551114. PMID: 28698369 PMC5547636

[74] Clement RL Daccache J Mohammed MT Diallo A Blazar BR Kuchroo BK . Follicular regulatory T cells control humoral and allergic immunity by restraining early B cell responses. Nat Immunol. (2019) 20:1360–71. doi: 10.1038/s41590-019-0472-4. PMID: 31477921 PMC6754271

[75] Laidlaw BJ Lu Y Amezquita RA Weinstein JS Vander Heiden JA Gupta NT . Interleukin-10 from CD4+ follicular regulatory T cells promotes the germinal center response. Sci Immunol. (2017) 2:eaan4767. doi: 10.1126/sciimmunol.aan4767. PMID: 29054998 PMC5846620

[76] Vella LA Buggert M Manne S Herati RS Sayin I Kuri-Cervantes L . T follicular helper cells in human efferent lymph retain lymphoid characteristics. J Clin Invest. (2019) 129:3185–200. doi: 10.1172/jci125628. PMID: 31264971 PMC6668682

[77] Sage PT Alvarez D Godec J von Andrian UH Sharpe AH . Circulating T follicular regulatory and helper cells have memory-like properties. J Clin Invest. (2014) 124:5191–204. doi: 10.1172/JCI76861. PMID: 25347469 PMC4348955

[78] Botta D Fuller MJ Marquez-Lago TT Bachus H Bradley JE Weinmann AS . Dynamic regulation of T follicular regulatory cell responses by interleukin 2 during influenza infection. Nat Immunol. (2017) 18:1249–60. doi: 10.1038/ni.3837. PMID: 28892471 PMC5679073

[79] Sage PT Sharpe AH . T follicular regulatory cells. Immunol Rev. (2016) 271:246–59. doi: 10.1111/imr.12411. PMID: 27088919

[80] Aloulou M Carr EJ Gador M Bignon A Liblau RS Fazilleau N . Follicular regulatory T cells can be specific for the immunizing antigen and derive from naive T cells. Nat Commun. (2016) 7:10579. doi: 10.1038/ncomms10579. PMID: 26818004 PMC4738360

[81] Xie MM Chen Q Liu H Yang K Koh B Wu H . Follicular regulatory T cells inhibit the development of granzyme B–expressing B cells in the germinal center. J Clin Invest. (2020) 130:4192–205. doi: 10.1172/JCI132249. PMID: 32255767 PMC7324176

[82] Lin PH Hsiao PJ Pan CF Liu MT Wang JT Ching C . Association of vaccine-specific regulatory T cells with reduced antibody response to repeated influenza vaccination. Eur J Immunol. (2023) 53:e2350525. doi: 10.1002/eji.202350525. PMID: 37713727

[83] Moritzky SA Richards KA Glover MA Krammer F Chaves FA Topham DJ . The negative effect of preexisting immunity on influenza vaccine responses transcends the impact of vaccine formulation type and vaccination history. J Infect Dis. (2023) 227:381–90. doi: 10.1093/infdis/jiac068. PMID: 35199825 PMC9891420

[84] Garten RJ Davis CT Russell CA Shu B Lindstrom S Balish A . Antigenic and genetic characteristics of swine-origin 2009 A(H1N1) influenza viruses circulating in humans. Science. (2009) 325:197–201. doi: 10.1126/science.1176225 19465683 PMC3250984

[85] Tong S Zhu X Li Y Shi M Zhang J Bourgeois M . New World bats harbor diverse influenza A viruses. PloS Pathog. (2013) 9:e1003657. doi: 10.1371/journal.ppat.1003657. PMID: 24130481 PMC3794996

[86] Krammer F Smith GJD Fouchier RAM Peiris M Kedzierska K Doherty PC . Influenza. Nat Rev Dis Primers. (2018) 4:3. doi: 10.1038/s41572-018-0002-y. PMID: 29955068 PMC7097467

[87] Tanner AR Dorey RB Brendish NJ Clark TW . Influenza vaccination: protecting the most vulnerable. Eur Respir Rev. (2021) 30:200258. doi: 10.1183/16000617.0258-2020. PMID: 33650528 PMC9488965

[88] Rondy M Kissling E Emborg H-D Gherasim A Pebody R Trebbien R . Interim 2017/18 influenza seasonal vaccine effectiveness: combined results from five European studies. Euro Surveill. (2018) 23:18–00086. doi: 10.2807/1560-7917.ES.2018.23.9.18-00086. PMID: 29510782 PMC5840921

[89] Zambon M Hayden FG . Influenza A(H3N2) subclade K virus: threat and response. JAMA. (2026) 335:307–10. doi: 10.1001/jama.2025.25903. PMID: 41411120

[90] Xu X Hull W Plunkett D Tu ZJ Ross TM Rhoads DD . Emergence of influenza A(H3N2) subclade K in northeast Ohio in autumn 2025. J Clin Microbiol. (2026) 64:e01813-25. doi: 10.1128/jcm.01813-25. PMID: 41589842 PMC12977577

[91] Quarleri J Delpino MV . Is the emerging influenza A(H3N2) K subclade a specific threat for older adults? Geroscience. (2026) 48:225–8. doi: 10.1007/s11357-026-02108-y. PMID: 41563712 PMC12972441

[92] Center for Disease Control and Prevention . 2025–2026 Flu Season. Available online at: https://www.cdc.gov/flu/season/2025-2026.html#:~:text=Circulating%20influenza%20viruses,countries%20in%20the%20Northern%20Hemisphere (Accessed April 29, 2026).

[93] Sullivan SG Poh XY Sanchez-Ovando S Hadiprodjo AJ Carolan L Chin YQ . Immunogenicity of high-dose recombinant influenza vaccine versus standard-dose egg-grown and cell-grown vaccines among frequently and infrequently vaccinated young adults in Singapore: a randomised, controlled, double-blind, single-centre, phase 4 clinical trial. Lancet Infect Dis. (2026). doi: 10.1016/S1473-3099(26)00062-9 41936374

[94] Minor PD . Vaccines against seasonal and pandemic influenza and the implications of changes in substrates for virus production. Clin Infect Dis. (2010) 50:560–5. doi: 10.1086/650171. PMID: 20085485

[95] Khan AS Mallet L Blümel J Cassart J-P Knezevic I Ng SHS . Report of the third conference on next-generation sequencing for adventitious virus detection in biologics for humans and animals. Biologicals. (2023) 83:101696. doi: 10.1016/j.biologicals.2023.101696. PMID: 37478506 PMC10522920

[96] Baez M Palese P Kilbourne ED . Gene composition of high-yielding influenza vaccine strains obtained by recombination. J Infect Dis. (1980) 141:362–5. doi: 10.1093/infdis/141.3.362. PMID: 7365284

[97] Kilbourne ED Murphy JS . Genetic studies of influenza viruses. I. Viral morphology and growth capacity as exchangeable genetic traits. Rapid in ovo adaptation of early passage Asian strain isolates by combination with PR8. J Exp Med. (1960) 111:387–406. doi: 10.1084/jem.111.3.387. PMID: 13755924 PMC2137265

[98] Parker L Wharton SA Martin SR Cross K Lin Y Liu Y . Effects of egg adaptation on receptor-binding and antigenic properties of recent influenza A (H3N2) vaccine viruses. J Gen Virol. (2016) 97:1333–44. doi: 10.1099/jgv.0.000457. PMID: 26974849 PMC5394856

[99] Rogers GN Paulson JC . Receptor determinants of human and animal influenza virus isolates: differences in receptor specificity of the H3 hemagglutinin based on species of origin. Virology. (1983) 127:361–73. doi: 10.1016/0042-6822(83)90150-2. PMID: 6868370

[100] Skehel JJ Wiley DC . Receptor binding and membrane fusion in virus entry: the influenza hemagglutinin. Annu Rev Biochem. (2000) 69:531–69. doi: 10.1146/annurev.biochem.69.1.531. PMID: 10966468

[101] Raymond DD Stewart SM Lee J Ferdman J Bajic G Do KT . Influenza immunization elicits antibodies specific for an egg-adapted vaccine strain. Nat Med. (2016) 22:1465–9. doi: 10.1038/nm.4223. PMID: 27820604 PMC5485662

[102] Liang W Tan TJC Wang Y Lv H Sun Y Bruzzone R . Egg-adaptive mutations of human influenza H3N2 virus are contingent on natural evolution. PloS Pathog. (2022) 18:e1010875. doi: 10.1371/journal.ppat.1010875. PMID: 36155668 PMC9536752

[103] Liu F Gross FL Joshi S Gaglani M Naleway AL Murthy K . Redirecting antibody responses from egg-adapted epitopes following repeat vaccination with recombinant or cell culture-based versus egg-based influenza vaccines. Nat Commun. (2024) 15:254. doi: 10.1038/s41467-023-44551-x. PMID: 38177116 PMC10767121

[104] Wu NC Lv H Thompson AJ Wu DC Ng WWS Kadam RU . Preventing an antigenically disruptive mutation in egg-based H3N2 seasonal influenza vaccines by mutational incompatibility. Cell Host Microbe. (2019) 25:836–844.e5. doi: 10.1016/j.chom.2019.04.013. PMID: 31151913 PMC6579542

[105] Liu F Gross FL Jefferson SN Holiday C Bai Y Wang L . Age-specific effects of vaccine egg adaptation and immune priming on A(H3N2) antibody responses following influenza vaccination. J Clin Invest. (2021) 131:e146138. doi: 10.1172/JCI146138. PMID: 33690218 PMC8262463

[106] Katz JM Webster RG . Amino acid sequence identity between the HA1 of influenza A (H3N2) viruses grown in mammalian and primary chick kidney cells. J Gen Virol. (1992) 73:1159–65. doi: 10.1099/0022-1317-73-5-1159. PMID: 1588320

[107] Schild GC Oxford JS de Jong JC Webster RG . Evidence for host-cell selection of influenza virus antigenic variants. Nature. (1983) 303:706–9. doi: 10.1038/303706a0. PMID: 6190093

[108] Harding AT Heaton NS . Efforts to improve the seasonal influenza vaccine. Vaccines. (2018) 6. doi: 10.3390/vaccines6020019. PMID: 29601497 PMC6027170

[109] Wu NC Zost SJ Thompson AJ Oyen D Nycholat CM McBride R . A structural explanation for the low effectiveness of the seasonal influenza H3N2 vaccine. PloS Pathog. (2017) 13:e1006682. doi: 10.1371/journal.ppat.1006682. PMID: 29059230 PMC5667890

[110] Oxford JS Newman R Corcoran T Bootman J Major D Yates P . Direct isolation in eggs of influenza A (H1N1) and B viruses with haemagglutinins of different antigenic and amino acid composition. J Gen Virol. (1991) 72:185–9. doi: 10.1099/0022-1317-72-1-185. PMID: 1990062

[111] Kishida D Fujisaki S Yokoyama M Sato H Saito R Ikematsu H . Evaluation of influenza virus A/H3N2 and B vaccines on the basis of cross reactivity of postvaccination human serum antibodies against influenza viruses A/H3N2 and B isolated in MDCK cells and embryonated hen eggs. Clin Vaccine Immunol. (2012) 19:897–908. doi: 10.1128/CVI.05726-11. PMID: 22492743 PMC3370437

[112] Robertson JS Nicolson C Major D Robertson EW Wood JM . The role of amniotic passage in the egg-adaptation of human influenza virus is revealed by haemagglutinin sequence analyses. J Gen Virol. (1993) 74:2047–51. doi: 10.1099/0022-1317-74-10-2047. PMID: 8409929

[113] Kodihalli S Justewicz DM Gubareva LV Webster RG . Selection of a single amino acid substitution in the hemagglutinin molecule by chicken eggs can render influenza A virus (H3) candidate vaccine ineffective. J Virol. (1995) 69:4888–97. doi: 10.1128/JVI.69.8.4888-4897.1995. PMID: 7609057 PMC189303

[114] Katz JM Webster RG . Efficacy of inactivated influenza A virus (H3N2) vaccines grown in mammalian cells or embryonated eggs. J Infect Dis. (1989) 160:191–8. doi: 10.1093/infdis/160.2.191. PMID: 2760480

[115] Robertson JS Bootman JS Newman R Oxford JS Daniels RS Webster RG . Structural changes in the haemagglutinin which accompany egg adaptation of an influenza A(H1N1) virus. Virology. (1987) 160:31–7. doi: 10.1016/0042-6822(87)90040-7. PMID: 3629978

[116] Rocha EP Xu X Hall HE Allen JR Regnery HL Cox NJ . Comparison of 10 influenza A (H1N1 and H3N2) haemagglutinin sequences obtained directly from clinical specimens to those of MDCK cell- and egg-grown viruses. J Gen Virol. (1993) 74:2513–8. doi: 10.1099/0022-1317-74-11-2513. PMID: 8245870

[117] Skowronski DM Janjua NZ De Serres G Sabaiduc S Eshaghi A Dickinson JA . Low 2012–13 influenza vaccine effectiveness associated with mutation in the egg-adapted H3N2 vaccine strain not antigenic drift in circulating viruses. PloS One. (2014) 9:e92153. doi: 10.1371/journal.pone.0092153. PMID: 24667168 PMC3965421

[118] McLean HQ Thompson MG Sundaram ME Kieke BA Gaglani M Murthy K . Influenza vaccine effectiveness in the United States during 2012–2013: variable protection by age and virus type. J Infect Dis. (2015) 211:1529–40. doi: 10.1093/infdis/jiu647. PMID: 25406334 PMC4407759

[119] Zost SJ Parkhouse K Gumina ME Kim K Perez SD Wilson PC . Contemporary H3N2 influenza viruses have a glycosylation site that alters binding of antibodies elicited by egg-adapted vaccine strains. Proc Natl Acad Sci USA. (2017) 114:12578–83. doi: 10.1073/pnas.1712377114. PMID: 29109276 PMC5703309

[120] Dunkle LM Izikson R Patriarca P Goldenthal KL Muse D Callahan J . Efficacy of recombinant influenza vaccine in adults 50 years of age or older. New Engl J Med. (2017) 376:2427–36. doi: 10.1056/NEJMoa1608862. PMID: 28636855

[121] Scorza FB Tsvetnitsky V Donnelly JJ . Universal influenza vaccines: Shifting to better vaccines. Vaccine. (2016) 34:2926–33. doi: 10.1016/j.vaccine.2016.03.085. PMID: 27038130 PMC4899887

[122] Rajaram S Wojcik R Moore C de Lejarazu RO de Lusignan S Montomoli E . The impact of candidate influenza virus and egg-based manufacture on vaccine effectiveness: Literature review and expert consensus. Vaccine. (2020) 38:6047–56. doi: 10.1016/j.vaccine.2020.06.021. PMID: 32600916

[123] Allen JD Ross TM . H3N2 influenza viruses in humans: Viral mechanisms, evolution, and evaluation. Hum Vaccines Immunotherapeutics. (2018) 14:1840–7. doi: 10.1080/21645515.2018.1462639. PMID: 29641358 PMC6149781

[124] Prasai K Yang Z Guan M Li T Ware D Hang J . Intrahost HA polymorphisms and culture adaptation shape antigenic profiles of H3N2 influenza viruses. J Virol. (2026) 100:e01775-25. doi: 10.1128/jvi.01775-25. PMID: 41498543 PMC12831994

[125] Rudman Spergel AK Wu I Deng W Cardona J Johnson K Espinosa-Fernandez I . Immunogenicity and safety of influenza and COVID-19 multicomponent vaccine in adults ≥50 years: A randomized clinical trial. JAMA. (2025) 333:1977–87. doi: 10.1001/jama.2025.5646. PMID: 40332892 PMC12060023

[126] Soens M Ananworanich J Hicks B Lucas KJ Cardona J Sher L . A phase 3 randomized safety and immunogenicity trial of mRNA-1010 seasonal influenza vaccine in adults. Vaccine. (2025) 50:126847. doi: 10.1016/j.vaccine.2025.126847. PMID: 39919447

[127] Henry C Makrinos D Liu R Cavallaro M Fenderson B Sun Y . An mRNA influenza vaccine induces immunity comparable to an adjuvanted vaccine in a randomized trial. NPJ Vaccines. (2026) 11:50. doi: 10.1038/s41541-026-01370-7. PMID: 41535296 PMC12901179

[128] Fitz-Patrick D McVinnie DS Jackson LA Crowther G Geevarughese A Cannon KD . Efficacy, immunogenicity, and safety of modified mRNA influenza vaccine. N Engl J Med. (2025) 393:2001–11. doi: 10.1056/NEJMoa2416779. PMID: 41259756

[129] Künzel W Glathe H Engelmann H Van Hoecke C . Kinetics of humoral antibody response to trivalent inactivated split influenza vaccine in subjects previously vaccinated or vaccinated for the first time. Vaccine. (1996) 14:1108–10. doi: 10.1016/0264-410x(96)00061-8. PMID: 8911005

[130] Smith DJ Forrest S Ackley DH Perelson AS . Variable efficacy of repeated annual influenza vaccination. Proc Natl Acad Sci USA. (1999) 96:14001–6. doi: 10.1073/pnas.96.24.14001. PMID: 10570188 PMC24180

[131] Carrat F Flahault A . Influenza vaccine: the challenge of antigenic drift. Vaccine. (2007) 25:6852–62. doi: 10.1016/j.vaccine.2007.07.027. PMID: 17719149

[132] Kim JH Skountzou I Compans R Jacob J . Original antigenic sin responses to influenza viruses. J Immunol. (2009) 183:3294–301. doi: 10.4049/jimmunol.0900398. PMID: 19648276 PMC4460008

[133] Skowronski DM Janjua NZ De Serres G Winter A-L Dickinson JA Gardy JL . A sentinel platform to evaluate influenza vaccine effectiveness and new variant circulation, Canada 2010–2011 season. Clin Infect Dis. (2012) 55:332–42. doi: 10.1093/cid/cis431. PMID: 22539661

[134] Ohmit SE Petrie JG Malosh RE Cowling BJ Thompson MG Shay DK . Influenza vaccine effectiveness in the community and the household. Clin Infect Dis. (2013) 56:1363–9. doi: 10.1093/cid/cit060. PMID: 23413420 PMC3693492

[135] Sullivan SG Kelly H . Stratified estimates of influenza vaccine effectiveness by prior vaccination: caution required. Clin Infect Dis. (2013) 57:474–6. doi: 10.1093/cid/cit255 23619811

[136] Ohmit SE Thompson MG Petrie JG Thaker SN Jackson ML Belongia EA . Influenza vaccine effectiveness in the 2011–2012 season: protection against each circulating virus and the effect of prior vaccination on estimates. Clin Infect Dis. (2014) 58:319–27. doi: 10.1093/cid/cit736. PMID: 24235265 PMC4007111

[137] McLean HQ Thompson MG Sundaram ME Meece JK McClure DL Friedrich TC . Impact of repeated vaccination on vaccine effectiveness against influenza A(H3N2) and B during 8 seasons. Clin Infect Dis. (2014) 59:1375–85. doi: 10.1093/cid/ciu680. PMID: 25270645 PMC4207422

[138] Skowronski DM Janjua NZ Sabaiduc S De Serres G Wintr A-L Gubbay JB . Influenza A/subtype and B/lineage effectiveness estimates for the 2011–2012 trivalent vaccine: cross-season and cross-lineage protection with unchanged vaccine. J Infect Dis. (2014) 210:126–37. doi: 10.1093/infdis/jiu048. PMID: 24446529

[139] Syrjänen RK Jokinen J Ziegler T Sundman J Lahdenkari M Julkunen I . Effectiveness of pandemic and seasonal influenza vaccines in preventing laboratory-confirmed influenza in adults: a clinical cohort study during epidemic seasons 2009–2010 and 2010–2011 in Finland. PloS One. (2014) 9:e108538. doi: 10.1371/journal.pone.0108538. PMID: 25265186 PMC4180439

[140] Skowronski DM Chambers C Sabaiduc S de Serres G Winter A-L Dickinson JA . Integrated sentinel surveillance linking genetic, antigenic, and epidemiologic monitoring of influenza vaccine-virus relatedness and effectiveness during the 2013–2014 influenza season. J Infect Dis. (2015) 212:726–39. doi: 10.1093/infdis/jiv177. PMID: 25784728

[141] Fu C Xu J Lin J Wang M Li K Ge J . Concurrent and cross-season protection of inactivated influenza vaccine against A(H1N1)pdm09 illness among young children: 2012–2013 case-control evaluation of influenza vaccine effectiveness. Vaccine. (2015) 33:2917–21. doi: 10.1016/j.vaccine.2015.04.063 25921713

[142] Bedford T Riley S Barr IG Broor S Chadha M Cox NJ . Global circulation patterns of seasonal influenza viruses vary with antigenic drift. Nature. (2015) 523:217–20. doi: 10.1038/nature14460 PMC4499780 26053121

[143] Skowronski DM Chambers C Sabaiduc S De Serres G Dickinson JA Winter AL . Interim estimates of 2014/15 vaccine effectiveness against influenza A(H3N2) from Canada’s Sentinel Physician Surveillance Network, January 2015. Euro Surveill. (2015) 20:21022. doi: 10.2807/1560-7917.es2015.20.4.21022 25655053

[144] Valenciano M Kissling E Reuss A Rizzo C Gherasim A Horváth JK . Vaccine effectiveness in preventing laboratory-confirmed influenza in primary care patients in a season of co-circulation of influenza A(H1N1)pdm09, B and drifted A(H3N2), I-MOVE multicentre case–control study, Europe 2014/15. Euro Surveill. (2016) 21. doi: 10.2807/1560-7917.ES.2016.21.7.30139. PMID: 26924024

[145] Skowronski DM Chambers C Sabaiduc S De Serres G Winter AL Dickinson JA . A perfect storm: impact of genomic variation and serial vaccination on low influenza vaccine effectiveness during the 2014–2015 season. Clin Infect Dis. (2016) 63:21–32. doi: 10.1093/cid/ciw176. PMID: 27025838 PMC4901864

[146] Skowronski DM Chambers C De Serres G Sabaiduc S Winter A-L Dickinson JA . Serial vaccination and the antigenic distance hypothesis: effects on influenza vaccine effectiveness during A(H3N2) epidemics in Canada, 2010–2011 to 2014–2015. J Infect Dis. (2017) 215:1059–69. doi: 10.1093/infdis/jix074. PMID: 28180277 PMC5853783

[147] Skowronski DM Chambers C Sabaiduc S De Serres G Winter AL Dickinson JA . Beyond antigenic match: possible agent-host and immuno-epidemiological influences on influenza vaccine effectiveness during the 2015–2016 season in Canada. J Infect Dis. (2017) 216:1487–500. doi: 10.1093/infdis/jix526. PMID: 29029166 PMC5853508

[148] Sullivan SG Chilver MB Carville KS Deng Y-M Grant KA Higgins G . Low interim influenza vaccine effectiveness, Australia, 1 May to 24 September 2017. Euro Surveill. (2017) 22:17–00707. doi: 10.2807/1560-7917.ES.2017.22.43.17-00707. PMID: 29090681 PMC5718387

[149] Rondy M El Omeiri N Thompson MG Leveque A Moren A Sullivan SG . Effectiveness of influenza vaccines in preventing severe influenza illness among adults: a systematic review and meta-analysis of test-negative design case–control studies. J Infect. (2017) 75:381–94. doi: 10.1016/j.jinf.2017.09.010. PMID: 28935236 PMC5912669

[150] Saito N Komori K Suzuki M Morimoto K Kishikawa T Yasaka T . Negative impact of prior influenza vaccination on current influenza vaccination among people infected and not infected in prior season: a test-negative case–control study in Japan. Vaccine. (2017) 35:687–93. doi: 10.1016/j.vaccine.2016.11.024. PMID: 28043738

[151] Bartoszko JJ McNamara IF Aras OAZ Hylton DA Zhang YB Malhotra D . Does consecutive influenza vaccination reduce protection against influenza: a systematic review and meta-analysis. Vaccine. (2018) 36:3434–44. doi: 10.1016/j.vaccine.2018.04.049. PMID: 29724509

[152] Ferdinands JM Fry AM Reynolds S Petrie J Flannery B Jackson ML . Intraseason waning of influenza vaccine protection: evidence from the US Influenza Vaccine Effectiveness Network, 2011–12 through 2014–15. Clin Infect Dis. (2017) 64:544–50. doi: 10.1093/cid/ciw816 28039340

[153] Saito N Komori K Suzuki M Kishikawa T Yasaka T Ariyoshi K . Dose-dependent negative effects of prior multiple vaccinations against influenza A and influenza B among schoolchildren: a study of Kamigoto Island in Japan during the 2011–2012, 2012–2013, and 2013–2014 influenza seasons. Clin Infect Dis. (2018) 67:897–904. doi: 10.1093/cid/ciy202. PMID: 29528389

[154] Kissling E Pozo F Buda S Vilcu A-M Gherasim A Brytting M . Low 2018/19 vaccine effectiveness against influenza A(H3N2) among 15-64-year-olds in Europe: exploration by birth cohort. Euro Surveill. (2019) 24:1900604. doi: 10.2807/1560-7917.ES.2019.24.48.1900604. PMID: 31796152 PMC6891946

[155] Skowronski DM Sabaiduc S Leir S Rose C Zou M Murti M . Paradoxical clade- and age-specific vaccine effectiveness during the 2018/19 influenza A(H3N2) epidemic in Canada: potential imprint-regulated effect of vaccine (I-REV). Euro Surveill. (2019) 24:1900585. doi: 10.2807/1560-7917.ES.2019.24.46.1900585. PMID: 31771709 PMC6864978

[156] Zhang A Stacey HD Mullarkey CE Miller MS . Original antigenic sin: how first exposure shapes lifelong anti-influenza virus immune responses. J Immunol. (2019) 202:335–40. doi: 10.4049/jimmunol.1801149. PMID: 30617114

[157] Kim SS Flannery B Foppa IM Chung JR Nowalk MP Zimmerman RK . Effects of prior season vaccination on current season vaccine effectiveness in the United States Flu Vaccine Effectiveness Network, 2012–2013 through 2017-2018. Clin Infect Dis. (2021) 73:497–505. doi: 10.1093/cid/ciaa706. PMID: 32505128 PMC8326585

[158] Okoli GN Racovitan F Abdulwahid T Hyder SK Lansbury L Righolt CH . Decline in seasonal influenza vaccine effectiveness with vaccination program maturation: a systematic review and meta-analysis. Open Forum Infect Dis. (2021) 8:ofab069. doi: 10.1093/ofid/ofab069. PMID: 33738320 PMC7953658

[159] Jones-Gray E Robinson EJ Kucharski AJ Fox A Sullivan SG . Does repeated influenza vaccination attenuate effectiveness? A systematic review and meta-analysis. Lancet Respir Med. (2023) 11:27–44. doi: 10.1016/S2213-2600(22)00266-1. PMID: 36152673 PMC9780123

[160] Hoskins TW Davies JR Smith AJ Miller CL Allchin A . Assessment of inactivated influenza-A vaccine after three outbreaks of influenza A at Christ’s Hospital. Lancet. (1979) 1:33–5. doi: 10.1016/s0140-6736(79)90468-9. PMID: 83475

[161] Mosterín Höpping A McElhaney J Fonville JM Powers DC Beyer WEP Smith DJ . The confounded effects of age and exposure history in response to influenza vaccination. Vaccine. (2016) 34:540–6. doi: 10.1016/j.vaccine.2015.11.058. PMID: 26667611 PMC4724805

[162] Beyer WE de Bruijn IA Palache AM Westendorp RG Osterhaus AD . Protection against influenza after annually repeated vaccination: a meta-analysis of serologic and field studies. Arch Intern Med. (1999) 159:182–8. doi: 10.1001/archinte.159.2.182. PMID: 9927102

[163] Nabeshima S Kashiwagi K Murata M Kanamoto Y Furusyo N Hayashi J . Antibody response to influenza vaccine in adults vaccinated with identical vaccine strains in consecutive years. J Med Virol. (2007) 79:320–5. doi: 10.1002/jmv.20801. PMID: 17245715

[164] Sasaki S He XS Holmes TH Dekker CL Kemble GW Arvin AM . Influence of prior influenza vaccination on antibody and B-cell responses. PloS One. (2008) 3:e2975. doi: 10.1371/journal.pone.0002975. PMID: 18714352 PMC2500171

[165] Huijskens E Rossen J Mulder P van Beek R van Vugt H Verbakel J . Immunogenicity, boostability, and sustainability of the immune response after vaccination against Influenza A virus (H1N1) 2009 in a healthy population. Clin Vaccine Immunol. (2011) 18:1401–5. doi: 10.1128/CVI.05046-11. PMID: 21795459 PMC3165238

[166] Thompson MG Naleway A Fry AM Ball S Spencer SM Reynolds S . Effects of repeated annual inactivated influenza vaccination among healthcare personnel on serum hemagglutinin inhibition antibody response to A/Perth/16/2009 (H3N2)-like virus during 2010–11. Vaccine. (2016) 34:981–8. doi: 10.1016/j.vaccine.2015.10.119. PMID: 26813801 PMC5218812

[167] Leung VKY Carolan LA Worth LJ Harper SA Peck H Tilmanis D . Influenza vaccination responses: evaluating impact of repeat vaccination among health care workers. Vaccine. (2017) 35:2558–68. doi: 10.1016/j.vaccine.2017.03.063. PMID: 28385605

[168] Leung VKY Fox A Carolan LA Aban M Laurie KL Druce J . Impact of prior vaccination on antibody response and influenza-like illness among Australian healthcare workers after influenza vaccination in 2016. Vaccine. (2021) 39:3270–8. doi: 10.1016/j.vaccine.2021.04.036. PMID: 33985853

[169] Fox A Carolan L Leung V Phuong HVM Khvorov A Auladell M . Opposing effects of prior infection versus prior vaccination on vaccine immunogenicity against influenza A(H3N2) viruses. Viruses. (2022) 14:470. doi: 10.3390/v14030470. PMID: 35336877 PMC8949461

[170] Petrie JG Ohmit SE Johnson E Truscon R Monto AS . Persistence of antibodies to influenza hemagglutinin and neuraminidase following one or two years of influenza vaccination. J Infect Dis. (2015) 212:1914–22. doi: 10.1093/infdis/jiv313. PMID: 26014800 PMC4655854

[171] Sullivan SG Khvorov A Carolan L Dowson L Hadiprodjo AJ Sánchez-Ovando S . Antibody responses against influenza A decline with successive years of annual influenza vaccination. NPJ Vaccines. (2025) 10. doi: 10.1038/s41541-024-01057-x. PMID: 39820465 PMC11739582

[172] Auladell M Phuong HVM Mai LTQ Tseng Y-Y Carolan L Wilks S . Influenza virus infection history shapes antibody responses to influenza vaccination. Nat Med. (2022) 28:363–72. doi: 10.1038/s41591-022-01690-w. PMID: 35177857

[173] Davenport FM Hennessy AV Stuart-Harris CH Francis T . Epidemiology of influenza; comparative serological observations in England and the United States. Lancet. (1955) 266:469–74. doi: 10.1016/s0140-6736(55)93328-6. PMID: 13252891

[174] Davenport FM Hennessy AV Francis T . Epidemiologic and immunologic significance of age distribution of antibody to antigenic variants of influenza virus. J Exp Med. (1953) 98:641–56. doi: 10.1084/jem.98.6.641. PMID: 13109114 PMC2136340

[175] Henry C Palm AE Krammer F Wilson PC . From original antigenic sin to the universal influenza virus vaccine. Trends Immunol. (2018) 39:70–9. doi: 10.1016/j.it.2017.08.003. PMID: 28867526 PMC5748348

[176] Lessler J Riley S Read JM Wang S Zhu H Smith GJD . Evidence for antigenic seniority in influenza A (H3N2) antibody responses in southern China. PloS Pathog. (2012) 8:e1002802. doi: 10.1371/journal.ppat.1002802. PMID: 22829765 PMC3400560

[177] Sanyal M Holmes TH Maecker H Albretch RA Dekker CL He X-S . Diminished B-cell response after repeat influenza vaccination. J Infect Dis. (2019) 219:1586–95. doi: 10.1093/infdis/jiy685. PMID: 30496437 PMC6473172

[178] Zhang M Ma J Li M . Original antigenic sin in CD4+ T cells. Immunology. (2025) 175:165–79. doi: 10.1111/imm.13916. PMID: 40056013

[179] Klenerman P Zinkernagel RM . Original antigenic sin impairs cytotoxic T lymphocyte responses to viruses bearing variant epitopes. Nature. (1998) 394:482–5. doi: 10.1038/28860. PMID: 9697771

[180] Choi YS Baek YH Kang W Nam SJ Lee J You S . Reduced antibody responses to the pandemic (H1N1) 2009 vaccine after recent seasonal influenza vaccination. Clin Vaccine Immunol. (2011) 18:1519–23. doi: 10.1128/CVI.05053-11. PMID: 21813667 PMC3165229

[181] Hirst GK . The quantitative determination of influenza virus and antibodies by means of red cell agglutination. J Exp Med. (1942) 75:49–64. doi: 10.1084/jem.75.1.49. PMID: 19871167 PMC2135212

[182] Wood JM Gaines-Das RE Taylor J Chakraverty P . Comparison of influenza serological techniques by international collaborative study. Vaccine. (1994) 12:167–74. doi: 10.1016/0264-410X(94)90056-6. PMID: 8147099

[183] Wood JM Major D Heath A Newman RW Hoschler K Stephenson I . Reproducibility of serology assays for pandemic influenza H1N1: Collaborative study to evaluate a candidate WHO International Standard. Vaccine. (2012) 30:210–7. doi: 10.1016/j.vaccine.2011.11.019. PMID: 22100887

[184] Wilson G Ye Z Xie H Vahl S Dawson E Rowlen K . Automated interpretation of influenza hemagglutination inhibition (HAI) assays: Is plate tilting necessary? PloS One. (2017) 12:e0179939. doi: 10.1371/journal.pone.0179939. PMID: 28662088 PMC5491073

[185] Waldock J Zheng L Remarque EJ Civet A Hu B Jalloh SL . Assay harmonization and use of biological standards to improve the reproducibility of the hemagglutination inhibition assay: a FLUCOP collaborative study. mSphere. (2021) 6:e0056721. doi: 10.1128/mSphere.00567-21. PMID: 34319129 PMC8530177

[186] Waldock J Remarque EJ Zheng L Ho S Hoschler K Neumann B . Haemagglutination inhibition and virus microneutralisation serology assays: use of harmonised protocols and biological standards in seasonal influenza serology testing and their impact on inter-laboratory variation and assay correlation: a FLUCOP collaborative study. Front Immunol. (2023) 14:1155552. doi: 10.3389/fimmu.2023.1155552. PMID: 37143658 PMC10151801

[187] Dunning AJ DiazGranados CA Voloshen T Hu B Landolfi VA Talbot HK . Correlates of protection against influenza in the elderly: results from an influenza vaccine efficacy trial. Clin Vaccine Immunol. (2016) 23:228–35. doi: 10.1128/CVI.00604-15. PMID: 26762363 PMC4783426

[188] Zhang X Ross TM . Anti-neuraminidase immunity in the combat against influenza. Expert Rev Vaccines. (2024) 23:474–84. doi: 10.1080/14760584.2024.2343689. PMID: 38632930 PMC11157429

[189] Weiss CD Wang W Lu Y Billings M Eick-Cost A Couzens L . Neutralizing and neuraminidase antibodies correlate with protection against influenza during a late season A/H3N2 outbreak among unvaccinated military recruits. Clin Infect Dis. (2020) 71:3096–102. doi: 10.1093/cid/ciz1198. PMID: 31840159 PMC7819518

[190] Walz L Kays S-K Zimmer G von Messling V . Neuraminidase-inhibiting antibody titers correlate with protection from heterologous influenza virus strains of the same neuraminidase subtype. J Virol. (2018) 92. doi: 10.1128/jvi.01006-18. PMID: 29925654 PMC6096819

[191] Eichelberger MC Monto AS . Neuraminidase, the forgotten surface antigen, emerges as an influenza vaccine target for broadened protection. J Infect Dis. (2019) 219:S75–80. doi: 10.1093/infdis/jiz017. PMID: 30715357 PMC7325326

[192] Assal OE Hall A Pangonis S Remien K Ulicny AK . The impact of COVID-19 pandemic social distancing and mask mandates on the prevalence of influenza and RSV during their peak season. Meeting Abstracts February 2022. Pediatrics. (2022) 149:193.

[193] Noh JY Seong H Yoon JG Song JY Cheong HJ Kim WJ . Social distancing against COVID-19: implications for the control of influenza. J Korean Med Sci. (2020) 35:e182. doi: 10.3346/jkms.2020.35.e182. PMID: 32419400 PMC7234863

[194] Ilari K Miia A Lotta M Katri B Tarja H-K Marjo R . Effect of social distancing due to the COVID-19 pandemic on the incidence of viral respiratory tract infections in children in Finland during early 2020. Ped Infect Dis J. (2020) 39:e423–7. doi: 10.1097/INF.0000000000002845. PMID: 32773660

[195] Centers for Disease Control and Prevention . Treating Flu with Antiviral Drugs, CDC (2024). Available online at: https://www.cdc.gov/flu/treatment/antiviral-drugs.html (Accessed April 29, 2026).

[196] Frutos AM Ahmad HM Ujamaa D O’Halloran AC Englund JA Klein EJ . Underutilization of influenza antiviral treatment among children and adolescents at higher risk for influenza-associated complications — United States, 2023–2024. MMWR Morb Mortal Wkly Rep. (2024) 73:1022–9. doi: 10.15585/mmwr.mm7345a2. PMID: 39541236 PMC11576051

[197] Hayden FG Whitley RJ . Introduction and update: advances in influenza therapeutics. J Infect Dis. (2025) 232:S169–76. doi: 10.1093/infdis/jiaf298. PMID: 41102604

[198] LaForce C Man CY Henderson FW McEIhaney JE Hampel Jr FC Bettis R . Efficacy and safety of inhaled zanamivir in the prevention of influenza in community-dwelling, high-risk adult and adolescent subjects: a 28-day, multicenter, randomized, double-blind, placebo-controlled trial. Clin Ther. (2007) 29:1579–90. doi: 10.1016/j.clinthera.2007.08.023. PMID: 17919541

[199] Dobson J Whitley RJ Pocock S Monto AS . Oseltamivir treatment for influenza in adults: a meta analysis of randomised controlled trials. Lancet. (2015) 385:1729–37. doi: 10.1016/S0140-6736(14)62449-1. PMID: 25640810

[200] Cowling BJ Lin Y Ikematsu H . Use of influenza antivirals to prevent transmission. J Infect Dis. (2025) 232:S215–26. doi: 10.1093/infdis/jiaf116. PMID: 41102613

[201] Gupta RK . The vital role of biological standardization in ensuring efficacy and safety of biological products - historical perspectives. J Pharm Sci. (2025) 114:690–700. doi: 10.1016/j.xphs.2024.12.011. PMID: 39710320

[202] Lyu X Zhao Q Hui J Wang T Lin M Wang K . The global landscape of approved antibody therapies. Antib Ther. (2022) 5:233–57. doi: 10.1093/abt/tbac021. PMID: 36213257 PMC9535261

[203] Liu JK . The history of monoclonal antibody development - progress, remaining challenges and future innovations. Ann Med Surg (Lond). (2014) 3:113–6. doi: 10.1016/j.amsu.2014.09.001. PMID: 25568796 PMC4284445

[204] Tan KW Ji P Qian Z Gao Q Wang S Li Q . Rapidly accelerated development of neutralizing COVID-19 antibodies by reducing cell line and CMC development timelines. Biotechnol Bioeng. (2025) 122:2287–96. doi: 10.1002/bit.28302. PMID: 36482495 PMC9877800

[205] McCafferty J Griffiths AD Winter G Chiswell DJ . Phage antibodies: filamentous phage displaying antibody variable domains. Nature. (1990) 348:552–4. doi: 10.1038/348552a0. PMID: 2247164

[206] Breitling F Dübel S Seehaus T Klewinghaus I Little M . A surface expression vector for antibody screening. Gene. (1991) 104:147–53. doi: 10.1016/0378-1119(91)90244-6. PMID: 1916287

[207] Barbas CF Kang AS Lerner RA Benkovic SJ . Assembly of combinatorial antibody libraries on phage surfaces: the gene III site. Proc Natl Acad Sci. (1991) 88:7978–82. doi: 10.1073/pnas.88.18.7978. PMID: 1896445 PMC52428

[208] Alfaleh MA Alsaab HO Mahmoud AB Alkayyal AA Jones ML Mahler SM . Phage display derived monoclonal antibodies: from bench to bedside. Front Immunol. (2020) 11:1986. doi: 10.3389/fimmu.2020.01986. PMID: 32983137 PMC7485114

[209] Hutchings CJ Sato AK . Phage display technology and its impact on the discovery of novel protein-based drugs. Expert Opin Drug Discov. (2024) 19:887–915. doi: 10.1080/17460441.2024.2367023. PMID: 39074492

[210] Crescioli S Kaplon H Wang L Visweswaraiah J Kapoor V Reichert JM . Antibodies to watch in 2025. mAbs. (2024) 17. doi: 10.1080/19420862.2024.2443538. PMID: 39711140 PMC12952251

[211] Klotchenko S Plotnikova M . mRNA-encoded antibodies: an emerging paradigm in antiviral protection. Biomolecules. (2026) 16:297. doi: 10.3390/biom16020297. PMID: 41750365 PMC12938726

[212] Singh D . mRNA-encoded antibodies as a next-generation therapeutic paradigm: a rapid and adaptive platform for the prevention and treatment of emerging and re-emerging infectious diseases - a critical review. Immunol Res. (2026) 74:7. doi: 10.1007/s12026-025-09737-z. PMID: 41521363

[213] Tkaczyk C Newton M Patnaik MM Thom G Strain M Gamson A . *In vivo* mRNA expression of a multi-mechanistic mAb combination protects against Staphylococcus aureus infection. Mol Ther. (2024) 32:2505–18. doi: 10.1016/j.ymthe.2024.05.036. PMID: 38822525 PMC11405172

[214] Vu MN Neil JA Mackenzie-Kludas C Kelley A Tan H-X Subbarao K . Delivery of monoclonal antibodies using mRNA lipid nanoparticles confers protection against SARS-CoV-2 and influenza. Mol Ther Nucleic Acids. (2026) 37. doi: 10.1016/j.omtn.2026.102873. PMID: 41858837 PMC12996773

[215] Food and Drug Administration . Platform technology designation program; draft guidance for industry; availability; agency information collection activities; proposed collection; comment request, federal register / vol. 89, no. 104 / Wednesday, May 29, 2024 / [Docket no. FDA–2024–D–1829. In: Federal Register. Washington DC: US Government Printing Office (GPO).

[216] FDA Guidance for Industry . Platform Technology Designation Program for Drug Development (2024). Available online at: https://www.fda.gov/regulatory-information/search-fda-guidance-documents/platform-technology-designation-program-drug-development (Accessed April 29, 2026).

[217] Simoes EA . Immunoprophylaxis of respiratory syncytial virus: global experience. Respir Res. (2002) 3:6. doi: 10.1186/rr187. PMID: 12119055 PMC1866370

[218] Tracy TJ Morabito KM Graham BS . Immunological lessons from respiratory syncytial virus vaccine development. Immunity. (2019) 51:429–42. doi: 10.1016/j.immuni.2019.08.007. PMID: 31533056

[219] Noor A Krilov LR . A historical perspective on respiratory syncytial virus prevention: a journey spanning over half a century from the setback of an inactive vaccine candidate to the success of passive immunization strategy. J Pediatr Infect Dis Soc. (2024) 13:S103–9. doi: 10.1093/jpids/piae027. PMID: 38577737

[220] Mahrous NN Alhumaidan OS Alkhoshaiban AS Tafish RT Al-Ghnnam FF Althubyani M . Broadly neutralizing monoclonal antibodies against influenza A viruses: current insights and future directions. Front Microbiol. (2026) 16:1738181. doi: 10.3389/fmicb.2025.1738181. PMID: 41602762 PMC12833332

[221] Hoy G Cortier T Maier HE Kuan G Lopez R Sanchez N . Anti-neuraminidase and anti-HA stalk antibodies reduce the susceptibility to and infectivity of influenza A/H3N2 virus. Nat Commun. (2025) 16:10910. doi: 10.1038/s41467-025-65283-0. PMID: 41381433 PMC12698684

[222] Momont C Dang HV Zatta F Hauser K Wang C di Lulio J . A pan-influenza antibody inhibiting neuraminidase via receptor mimicry. Nature. (2023) 618:590–7. doi: 10.1038/s41586-023-06136-y. PMID: 37258672 PMC10266979

[223] Lei R Kim W Lv H Mou Z Scherm MJ Schmitz AJ . Leveraging vaccination-induced protective antibodies to define conserved epitopes on influenza N2 neuraminidase. Immunity. (2023) 56:2621–34.e6. doi: 10.1016/j.immuni.2023.10.005. PMID: 37967533 PMC10655865

[224] Barberis I Myles P Ault SK Bragazzi NL Martini M . History and evolution of influenza control through vaccination: from the first monovalent vaccine to universal vaccines. J Prev Med Hyg. (2016) 57:E115–20. doi: 10.15167/2421-4248/jpmh2016.57.3.642. PMID: 27980374 PMC5139605

[225] Polack FP Thomas SJ Kitchin N Absalon J Gurtman A Lockhart S . Safety and efficacy of the BNT162b2 mRNA Covid-19 vaccine. N Engl J Med. (2020) 383:2603–15. doi: 10.1056/NEJMoa2034577. PMID: 33301246 PMC7745181

[226] Baden LR El Sahly HM Essink B Kotloff K Frey S Novak R . Efficacy and safety of the mRNA-1273 SARS-CoV-2 vaccine. N Engl J Med. (2021) 384:403–16. doi: 10.1056/NEJMoa2035389. PMID: 33378609 PMC7787219

[227] Pecetta S Rappuoli R . mRNA, the beginning of a new influenza vaccine game. Proc Natl Acad Sci. (2022) 119:e2217533119. doi: 10.1073/pnas.2217533119. PMID: 36469761 PMC9897424

[228] Ma N Xia ZW Zhang ZG Nian XX Li XD Gong Z . Development of an mRNA vaccine against a panel of heterologous H1N1 seasonal influenza viruses using a consensus hemagglutinin sequence. Emerg Microbes Infect. (2023) 12:2202278. doi: 10.1080/22221751.2023.2202278. PMID: 37067355 PMC10155637

[229] Sicard T Kassardjian A Julien JP . B cell targeting by molecular adjuvants for enhanced immunogenicity. Expert Rev Vaccines. (2020) 19:1023–39. doi: 10.1080/14760584.2020.1857736. PMID: 33252273

[230] Zhao T Cai Y Jiang Y He X Wei Y Yu Y . Vaccine adjuvants: mechanisms and platforms. Signal Transduct Target Ther. (2023) 8:283. doi: 10.1038/s41392-023-01557-7. PMID: 37468460 PMC10356842

[231] Grigoryan L Feng Y Bellusci L Lai L Wali B Ellis M . AS03 adjuvant enhances the magnitude, persistence, and clonal breadth of memory B cell responses to a plant-based COVID-19 vaccine in humans. Sci Immunol. (2024) 9:eadi8039. doi: 10.1126/sciimmunol.adi8039. PMID: 38579013 PMC11732256

[232] Coffman RL Sher A Seder RA . Vaccine adjuvants: putting innate immunity to work. Immunity. (2010) 33:492–503. doi: 10.1016/j.immuni.2010.10.002. PMID: 21029960 PMC3420356

[233] Mokalla VR Gundarapu S Kaushik RS Rajput M Tummala H . Influenza vaccines: current status, adjuvant strategies, and efficacy. Vaccines. (2025) 13:962. doi: 10.3390/vaccines13090962. PMID: 41012165 PMC12474428

[234] Boikos C Fischer L O'Brien D Vasey J Sylvester GC Mansi JA . Relative effectiveness of adjuvanted trivalent inactivated influenza vaccine versus egg-derived quadrivalent inactivated influenza vaccines and high-dose trivalent influenza vaccine in preventing influenza-related medical encounters in US adults ≥ 65 years during the 2017–2018 and 2018–2019 influenza seasons. Clin Infect Dis. (2021) 73:816–23. doi: 10.1093/cid/ciab152. PMID: 33605977 PMC8423477

[235] Lee GH Lim SG . CpG-adjuvanted hepatitis B vaccine (HEPLISAV-B®) update. Expert Rev Vaccines. (2021) 20:487–95. doi: 10.1080/14760584.2021.1908133. PMID: 33783302

[236] Cui L Zhang S Zeng Y Zhang L Lin M Hong M . Combination mRNA vaccine adjuvanted with CpG oligodeoxynucleotides enhances protection against respiratory virus infection. ACS Nano. (2026) 20:10872–88. doi: 10.1021/acsnano.5c14408. PMID: 41911534 PMC13085855

[237] Hoxie I Vasilev K Clark JJ Bushfield K Francis B Loganathan M . A recombinant N2 neuraminidase-based CpG 1018® adjuvanted vaccine provides protection against challenge with heterologous influenza viruses in mice and hamsters. Vaccine. (2024) 42:126269. doi: 10.1016/j.vaccine.2024.126269. PMID: 39241354

[238] Olson SM Newhams MM Halasa NB Feldstein LR Novak T Weiss SL . Vaccine effectiveness against life-threatening influenza illness in US children. Clin Infect Dis. (2022) 75:230–8. doi: 10.1093/cid/ciab931. PMID: 35024795

[239] Tenforde MW Kondor RJG Chung JR Zimmerman RK Nowalk MP Jackson ML . Effect of antigenic drift on influenza vaccine effectiveness in the United States-2019-2020. Clin Infect Dis. (2021) 73:e4244–50. doi: 10.1093/cid/ciaa1884. PMID: 33367650 PMC8664438

[240] Ranjeva S Subramanian R Fang VJ Leung GM Ip DKM Perera RAPM . Age-specific differences in the dynamics of protective immunity to influenza. Nat Commun. (2019) 10:1660. doi: 10.1038/s41467-019-09652-6. PMID: 30971703 PMC6458119

[241] Principi N Esposito S Gasparini R Marchisio P Crovari P Flu-Flu Study Group . Burden of influenza in healthy children and their households. Arch Dis Child. (2004) 89:1002–7. doi: 10.1136/adc.2003.045401corr1 PMC1719733 15499051

[242] Cauchemez S Valleron AJ Boëlle PY Flahault A Ferguson NM . Estimating the impact of school closure on influenza transmission from Sentinel data. In: Nature, vol. 452. (2008). 452:750–4. doi: 10.1038/nature06732 18401408

[243] Cauchemez S Ferguson NM Fox A Mai LQ Thanh LT Thai PQ . Determinants of influenza transmission in South East Asia: insights from a household cohort study in Vietnam. PloS Pathog. (2014) 10:e1004310. doi: 10.1371/journal.ppat.1004310. PMID: 25144780 PMC4140851

[244] Viboud C Boëlle PY Cauchemez S Lavenu A Valleron A-J Flahault A . Risk factors of influenza transmission in households. Br J Gen Pract. (2004) 54:684–9. doi: 10.1016/j.ics.2004.01.013. PMID: . PMID: 15353055; PMCID: PMC1326070. 15353055 PMC1326070

[245] Shrestha NK Burke PC Nowacki AS Gordon SM . Effectiveness of the influenza vaccine during the 2024–2025 respiratory viral season. medRxiv. (2025). doi: 10.1101/2025.01.30.25321421. PMID: 38621210

[246] Su Z Cheshmehzangi A McDonnell D da Veiga CP Xiang YT . Mind the "vaccine fatigue. Front Immunol. (2022) 13:839433. doi: 10.3389/fimmu.2022.839433. PMID: 35359948 PMC8960954

[247] Larson HJ Jarrett C Eckersberger E Smith DM Paterson P . Understanding vaccine hesitancy around vaccines and vaccination from a global perspective: a systematic review of published literature, 2007-2012. Vaccine. (2014) 32:2150–9. doi: 10.1016/j.vaccine.2014.01.081. PMID: 24598724

[248] Schmid P Rauber D Betsch C Lidolt G Denker M-L . Barriers of influenza vaccination intention and behavior – a systematic review of influenza vaccine hesitancy, 2005 – 2016. PloS One. (2017) 12. doi: 10.1371/journal.pone.0170550. PMID: 28125629 PMC5268454

[249] Chen F Jiang M Rabidoux S Robinson S . Public avoidance and epidemics: insights from an economic model. J Theor Biol. (2011) 278:107–19. doi: 10.1016/j.jtbi.2011.03.007 21419135

[250] Lyons-Weiler J . Inactivated influenza vaccine is a risk to public health. Available online at: https://www.globalresearch.ca/inactivated-influenza-vaccine-risk-public-health/5883829 (Accessed April 29, 2026).

[251] Reiber C Shattuck EC Fiore S Alperin P Davis V Moore J . Change in human social behavior in response to a common vaccine. Ann Epidemiol. (2010) 20:729–33. doi: 10.1016/j.annepidem.2010.06.014. PMID: 20816312

[252] Bayham J Kuminoff NV Gunn Q Fenichel EP . Measured voluntary avoidance behaviour during the 2009 A/H1N1 epidemic. Proc Biol Sci. (2015) 282:1–7. doi: 10.1098/rspb.2015.0814. PMID: 26511046 PMC4650148

[253] Fenichel EP Castillo-Chavez C Ceddia MG Chowell G Parra PAG Hickling GJ . Adaptive human behavior in epidemiological models. Proc Natl Acad Sci. (2011) 108:6306–11. doi: 10.1073/pnas.1011250108. PMID: 21444809 PMC3076845

[254] Springborn M Chowell G MacLachlan M Fenichel EP . Accounting for behavioral responses during a flu epidemic using home television viewing. BMC Infect Dis. (2015) 15:1–14. doi: 10.1186/s12879-016-1795-5. PMID: 25616673 PMC4304633

[255] Vilches TN Jaberi-Douraki M Moghadas SM . Risk of influenza infection with low vaccine effectiveness: the role of avoidance behaviour. Epidemiol Infection. (2019) 147:1–8:e75. doi: 10.1017/S0950268818003540. PMID: 30869007 PMC6518843

